# Echidna: integrated simulations of single-cell immune receptor repertoires and transcriptomes

**DOI:** 10.1093/bioadv/vbac062

**Published:** 2022-09-02

**Authors:** Jiami Han, Solène Masserey, Danielle Shlesinger, Raphael Kuhn, Chrysa Papadopoulou, Andreas Agrafiotis, Victor Kreiner, Raphael Dizerens, Kai-Lin Hong, Cédric Weber, Victor Greiff, Annette Oxenius, Sai T Reddy, Alexander Yermanos

**Affiliations:** Department of Biosystems Science and Engineering, ETH Zurich, Basel 4058, Switzerland; Department of Biosystems Science and Engineering, ETH Zurich, Basel 4058, Switzerland; Department of Biosystems Science and Engineering, ETH Zurich, Basel 4058, Switzerland; Department of Biosystems Science and Engineering, ETH Zurich, Basel 4058, Switzerland; Department of Biosystems Science and Engineering, ETH Zurich, Basel 4058, Switzerland; Department of Biosystems Science and Engineering, ETH Zurich, Basel 4058, Switzerland; Department of Biosystems Science and Engineering, ETH Zurich, Basel 4058, Switzerland; Department of Biosystems Science and Engineering, ETH Zurich, Basel 4058, Switzerland; Department of Biosystems Science and Engineering, ETH Zurich, Basel 4058, Switzerland; Department of Biosystems Science and Engineering, ETH Zurich, Basel 4058, Switzerland; Department of Immunology, University of Oslo, Oslo 0450, Norway; Institute of Microbiology, ETH Zurich, Zurich 8093, Switzerland; Department of Biosystems Science and Engineering, ETH Zurich, Basel 4058, Switzerland; Department of Biosystems Science and Engineering, ETH Zurich, Basel 4058, Switzerland; Institute of Microbiology, ETH Zurich, Zurich 8093, Switzerland; Department of Pathology and Immunology, University of Geneva, Geneva 1211, Switzerland

## Abstract

**Motivation:**

Single-cell sequencing now enables the recovery of full-length immune receptor repertoires [B cell receptor (BCR) and T cell receptor (TCR) repertoires], in addition to gene expression information. The feature-rich datasets produced from such experiments require extensive and diverse computational analyses, each of which can significantly influence the downstream immunological interpretations, such as clonal selection and expansion. Simulations produce validated standard datasets, where the underlying generative model can be precisely defined and furthermore perturbed to investigate specific questions of interest. Currently, there is no tool that can be used to simulate single-cell datasets incorporating immune receptor repertoires and gene expression.

**Results:**

We developed Echidna, an R package that simulates immune receptors and transcriptomes at single-cell resolution with user-tunable parameters controlling a wide range of features such as clonal expansion, germline gene usage, somatic hypermutation, transcriptional phenotypes and spatial location. Echidna can additionally simulate time-resolved B cell evolution, producing mutational networks with complex selection histories incorporating class-switching and B cell subtype information. We demonstrated the benchmarking potential of Echidna by simulating clonal lineages and comparing the known simulated networks with those inferred from only the BCR sequences as input. Finally, we simulated immune repertoire information onto existing spatial transcriptomic experiments, thereby generating novel datasets that could be used to develop and integrate methods to profile clonal selection in a spatially resolved manner. Together, Echidna provides a framework that can incorporate experimental data to simulate single-cell immune repertoires to aid software development and bioinformatic benchmarking of clonotyping, phylogenetics, transcriptomics and machine learning strategies.

**Availability and implementation:**

The R package and code used in this manuscript can be found at github.com/alexyermanos/echidna and also in the R package Platypus (Yermanos *et al.*, 2021). Installation instructions and the vignette for Echidna is described in the Platypus Computational Ecosystem (https://alexyermanos.github.io/Platypus/index.html). Publicly available data and corresponding sample accession numbers can be found in Supplementary Tables S2 and S3.

**Supplementary information:**

Supplementary data are available at *Bioinformatics Advances* online.

## 1. Introduction

The adaptive immune system plays a crucial role in the protection against a broad range of pathogens. The molecular recognition of foreign pathogens by B and T cells is orchestrated by their characteristic BCRs and TCRs, respectively. Sequence diversification is first introduced into BCRs and TCRs by the recombination of genomically encoded variable (V), diversity (D) and joining (J) gene segments (Tonegawa, 1983). This diversity is further increased by the combinatorial pairing of variable heavy (VH) and variable light (VL) chains for B cells and variable beta (Vb) and variable alpha (Va) chains for T cells. BCRs and their secreted form, antibodies, can undergo additional diversification through somatic hypermutation (SHM) (Diaz and Casali, 2002). Together, these processes result in a vast diversity (∼10^18^ and 10^13^ for human B and T cells, respectively), which provides the baseline interaction space that accommodates a seemingly infinite number of foreign antigens (Greiff *et al.*, 2017; Murphy and Weaver, 2016).

Advances in deep sequencing have provided the opportunity to quantify and map sequence diversities of immune repertoires. Indeed, repertoire sequencing has been instrumental to investigate fundamental immunological principles in the context of disease, infection and immunization (Horns *et al.*, 2020; Neumeier *et al.*, 2022a; Yermanos *et al.*, 2021). However, prior to the emergence of single-cell sequencing, repertoire sequencing was performed on bulk (pooled) cell samples, which has a number of limitations: the inability to recover full-length, paired sequences (VH + VL for B cells and Vb + Va for T cells), the reliance upon read counts as a proxy for clonal expansion, and the lack of individual cell phenotyping based on surface marker expression (Brown *et al.*, 2019; Georgiou *et al.*, 2014; Miho *et al.*, 2018; Rosati *et al.*, 2017; Yaari and Kleinstein, 2015). The advent of single-cell sequencing (scSeq) has revolutionized the resolution at which clonal selection of immune repertoires can be quantified (Friedensohn *et al.*, 2017). For example, scSeq workflows have been established that enable the simultaneous recovery of both full-length, paired BCR or TCR sequence (VDJ) and full transcriptome [gene expression (GEX)] information, thereby providing a crucial link between clonal selection and expansion with a high-dimensional transcriptional cell phenotype (Horns *et al.*, 2020; Khatun *et al.*, 2021; Lindeman *et al.*, 2018; Mathew *et al.*, 2021; Stubbington *et al.*, 2016; Yermanos *et al.*, 2021). Interpreting such datasets, however, remains challenging as the accompanying computational pipelines and software are still in their infancy (Borcherding *et al.*, 2020; Sturm *et al.*, 2020; Yermanos *et al.*, 2021). Although multiple tools have been developed to simulate immune receptors and single-cell transcriptomes (Davidsen *et al.*, 2019; Marcou *et al.*, 2018; Safonova *et al.*, 2015; Weber *et al.*, 2020; Yermanos *et al.*, 2017), these represent separate platforms and thus there remains a lack of software capable of simulating scSeq data of immune repertoires and transcriptomes. We therefore developed Echidna, an R package that simulates immune repertoires and their corresponding transcriptomes. Importantly, datasets are constructed in a style compatible with commonly used methods of scSeq (e.g. 10x Genomics). We furthermore demonstrated the applicability of Echidna by simulating immune repertoires with varying features such as cell phenotype, clonal expansion, SHM, antibody isotype and germline gene usage. Finally, we leveraged simulated and time-resolved B cell mutational networks and evaluated the accuracy when using clonal lineage reconstruction algorithms.

## 2. Methods

### 2.1 Simulating adaptive immune repertoires and gene expression profile at single-cell resolution

Immune receptor sequences were simulated by either appending germline gene segments together or using variational autoencoders trained on public repertoire data. Single-cell transcriptomes were simulated based on either user-supplied expression vectors or publicly available single-cell gene expression data. The final expression levels for an individual cell are based on sampling from the base vector in combination with a user-defined noise parameter. More detailed information on the generation of immune receptor sequences and corresponding gene expression profiles can be found in the Supplementary information.

### 2.2 Simulating SHM and inferring mutational networks

Echidna provides three methods to simulate SHM: ‘Poisson’, ‘Data-driven’ and ‘Motif’ methods, each of which sets distinct SHM occurrence probabilities across the V(D)J regions. Mutational networks were calculated by computing the pairwise edit distance from all unique antibody sequences within a clone and iteratively adding new sequences to the closest node in each network after initializing by the germline node. More detailed information is provided in the Supplementary Information.

### 2.3 Simulating immune receptor sequences for spatial transcriptomic data

Echidna provides three different methods to simulate immune receptor sequences with existing spatial transcriptomics datasets. This includes assigning cells based on: (i) random probabilities, (ii) B or T cell density or (iii) distance from germline. B cell evolutionary networks can similarly be visualized onto spatial images. More detailed methods can be found in the Supplementary Information.

## 3. Results

### 3.1 Echidna simulates time-resolved single-cell immune receptor repertoires

To address the lack of software capable of simulating integrated immune receptors and transcriptomes, we developed the R package Echidna. Echidna generates full-length and paired adaptive immune receptors with the corresponding gene expression information at single-cell resolution for both human and murine immune repertoires (Supplementary Fig. S1A). Importantly, the format of the resulting simulation mirrors the output files of the widely used 10x Genomics cellranger tool (Supplementary Fig. S1B), thereby rendering simulated data compatible with existing bioinformatics tools such as Platypus and Seurat (Satija *et al.*, 2015; Yermanos *et al.*, 2021).

The simulation first generates an initial repertoire of cells, each produced by a separate recombination event for both VDJ (VH for B cells, Vb for T cells) and VJ (VL for B cells, Va for T cells) chains. This can either be done as previously described (Yermanos *et al.*, 2017) by appending reference germline segments (referred to as ‘Appended’) from the Immunogenetics Database (IMGT) (Lefranc *et al.*, 2003) with diversity generated by insertions and deletions in the complementarity determining region 3 (CDR3) or by generating receptors sampled from a sequence pool generated by variational autoencoders (Friedensohn *et al.*, 2020) that were trained on publicly available BCR and TCR sequences from naive human and murine repertoires (Kovaltsuk *et al.*, 2018). In the case that a user wishes to generate an initial immune repertoire based on experimental data, it is additionally possible to supply custom receptor sequences as input.

Regardless of the selected V(D)J simulation method, the user can further specify whether these simulated initial sequences should be restricted to productive BCR and TCR sequences based on external alignment and annotations tools (Bolotin *et al.*, 2015). By specifying the starting repertoire size, which is defined by the number of B or T cell clones at the first simulated time step, the user can control the initial clonal diversity via simulated V(D)J recombination. Under default parameters, germline genes are uniformly sampled from the IMGT reference database and appended together with a probability of inserting or deleting nucleotides at the junctions (both VD and DJ for VH and Vb chains, and VJ for VL and Va chains) following a user-supplied probability distribution. While under default conditions, each germline gene has a uniform probability of being selected for V(D)J recombination, this distribution can be altered to preferentially utilize certain germline segments as is the case under physiological settings.

**Fig. 1.**
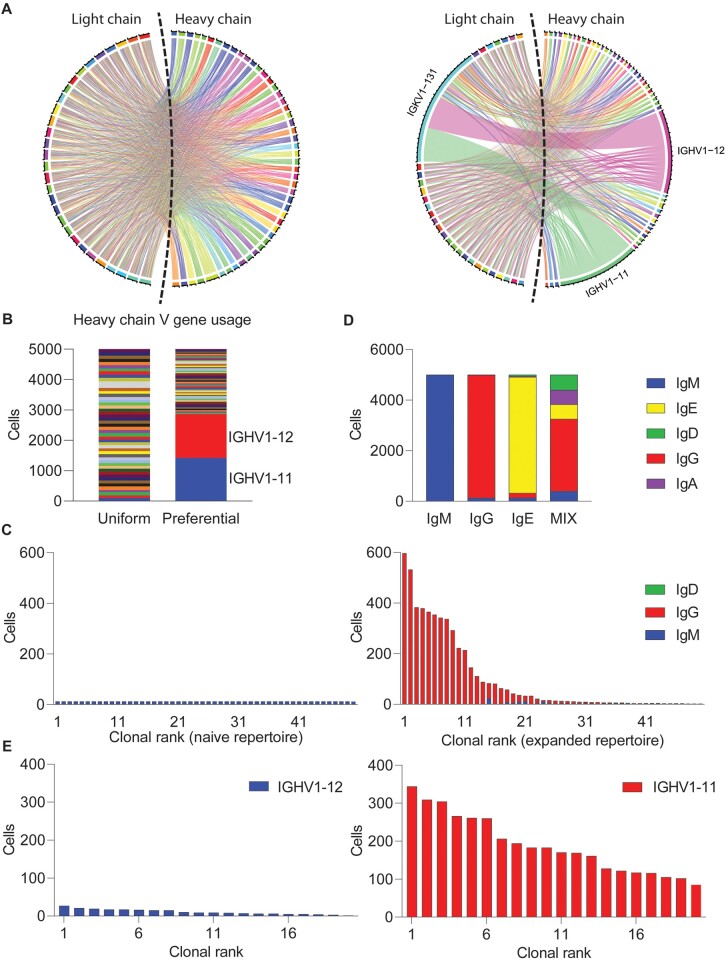
Echidna simulates expanded and naive immune repertoires at single-cell resolution. (**A**) Repertoires with either uniform or preferential germline gene usage of IGHV1-11. Each color represents a unique germline gene. Line width represents the number of cells using a particular variable heavy or variable light chain combination. (**B)** Heavy chain variable gene usage. Color indicates the unique segment. The two preferential germline genes used are specifically highlighted. (**C**) Clonal frequency for either naive-like (left) or expanded (right) repertoires. Clone is defined as all cells arising from a single simulated V(D)J recombination event. Bar color corresponds to the fraction of cells within each clone of a given isotype. (**D)** Isotype component of four simulations with different isotype switching probabilities. The bars represent the number of cells while the color indicates different isotypes. From left to right, the first three bars are repertoire dominated by IgM, IgG and IgE, while the last bar is repertoire simulated to have a mixture of all five isotypes. (**E**) Preferential selection and expansion based on germline V gene usage within a single repertoire. Clone is defined as all cells arising from a single simulated V(D)J recombination event.

To demonstrate the ability to simulate BCR repertoires, 5000 V(D)J recombination events were generated *in silico* where VH and VL genes were selected with either a uniform probability distribution or mirrored V gene distributions observed in experimental studies (Greiff *et al.*, 2017; Kräutler *et al.*, 2020; Yermanos *et al.*, 2020) (Fig. 1A). Such a preferential germline gene usage can also be utilized for V–J pairings of either the heavy or light chain, providing the user further control over the initial diversity generated by V(D)J recombination (Fig. 1B). In addition to the simplified random assortment of V, D and J germline genes, one can simulate the starting pool of immune receptors using variational autoencoders (VAEs), which have been recently used to identify convergent, antigen-specific antibodies and to generate novel sequences *in silico* (Friedensohn *et al.*, 2020). Both appending reference germline genes and using VAEs to generate starting V(D)J-recombined clones resulted in a high proportion of unproductive sequences (Supplementary Fig. S2A, B, C), which may not be ideal for downstream applications. Therefore, we have added the option to maintain only productive adaptive immune receptor sequences in the initial repertoire for either recombination option. To quantify sequence divergence between experimental and synthetic repertoires, we simulated sequences using both VAEs and Echidna’s appending method and subsequently calculated the Kullback–Leibler Divergence (KLD) based on position-wise nucleotide base frequencies for five pairs of groups: (i) experimental BCRs (training data) versus VAE-generated sequences, (ii) VAE-generated sequences versus Echidna’s naive V(D)J recombination (appending germline genes and random insertion/deletion at CDR3), (iii) Experimental BCRs (training data) versus randomly generated BCR sequences (each nucleotide position has a random chance for any of the four bases), (iv) VAE-generated sequences versus randomly generated BCR sequences (each nucleotide position has a random chance for any of the four bases) and (v) VAE-generated sequences versus Echidna’s naive V(D)J recombination (appending germline genes and random insertion/deletion at CDR3). We observed that Echidna’s method of appending germline genes and random insertion and deletion at junctions had comparable KLDs from experimental repertoires and were less than randomly generated antibody sequences (Supplementary Fig. S2D). It is likely, however, that further optimization or use of external tools capable of simulating V(D)J recombination (Davidsen *et al.*, 2019; Isacchini *et al.*, 2021; Marcou *et al.*, 2018; Sethna *et al.*, 2019, 2020) may have less divergence from experimental repertoires.

### 3.2 Repertoire features dictate simulated clonal expansion

Following initialization of the first cell for a given clone [defined by all cells arising from a unique V(D)J recombination event], the time-resolved phase of the simulation begins, where new cells can either be introduced into the repertoire, existing cells can undergo clonal expansion or cells can be removed from the simulation via cell death. Although the final simulated repertoire does not include the BCRs and TCRs of the cells that have undergone cell death, the history of these cells can still be analyzed and reconstructed by the internal storage of parameters such as adaptive immune receptor sequence. To demonstrate the ability of Echidna to simulate different modes of clonal expansion for B and T cells, we simulated either naive or expanded repertoires by altering the simulation parameters that control the probability of either undergoing cell division or recruiting new cells into the immune repertoire (Fig. 1C). The cell replication rate can increase or decrease at each time step proportionally to the current number of cells in the clone to which it belongs (Supplementary Fig. S4). This clone-level cell replication rate will finally influence the overall clonal distribution. In the case of B cells, cells can additionally undergo class-switch recombination from the starting IgM isotype with a user-supplied transition matrix. While the default transition matrix follows a probability distribution mirroring the biological distribution of class-switching (IgM > IgG > IgA > IgE), it is possible to alter this matrix to mirror cases where preferential class-switching occurs, such as viral infections or immunizations (Fig. 1D) (Neumeier *et al.*, 2022a, b). While the default settings of Echidna assign a uniform probability to undergo clonal expansion to all cells within a given clone (and SHM in the case of B cells), the user can further tune this parameter to link particular repertoire features, such as germline gene usage to clonal expansion, as visualized by certain clones which undergo preferential expansion (Fig. 1E). This is achieved by a ‘variant selection’ mechanism in Echidna, where variants (defined by a pair of unique VDJ and VJ sequence combinations) can be selected for by either (i) a random basis, (ii) a germline gene-determined manner or (iii) CDR3 length, which allows cells with the specific feature to acquire different expansion probabilities. The selected variants then maintain and transmit their expansion potential to newly generated cells that possess the same immune receptor sequence. In addition to assigning various clonal expansion rates, the user has the option to ensure that the selected cells have higher probabilities to undergo class-switch recombination and phenotype switching events. Taken together, these examples demonstrate the ability of Echidna to incorporate repertoire features, such as germline gene usage and isotype, into the model underlying clonal selection.

### 3.3 Time-resolved evolution of simulated B cell receptors

**Fig. 2.**
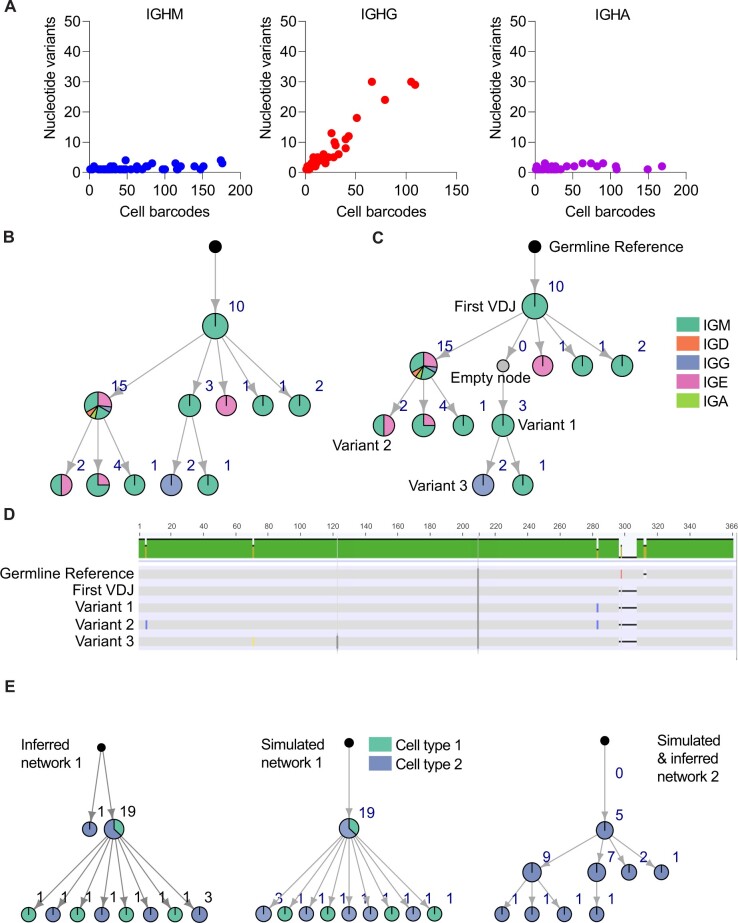
Simulation of time-resolved somatic hypermutation and evolution. (**A**) Relationship between the number of cells and the number of unique BCR sequences (variants) for each isotype. Each point represents a clone, defined as all cells belonging to a single simulated V(D)J recombination event. Variants are defined by a unique full-length VH–VL nucleotide sequence. (**B**) Simulated clonal lineages depicting the relationship between somatic hypermutation, clonal expansion and isotype. Each node depicts a unique variant, defined by a unique full-length VH–VL nucleotide sequence. The color of each node corresponds to the proportion of cells within that variant of a certain isotype. Edges depict clonal relationships. Black nodes correspond to unmutated reference germline. (**C**) Clonal lineage as in (B) but including the historical node that is no longer present by the end of the simulation due to mutation or cell death. (**D**) VH sequence alignment from the clonal lineage in (C). ‘Germline’ refers to the appended V, D and J reference genes without additional alterations to the CDR3. ‘First’ refers to the first simulated sequence following insertion and deletion during V(D)J recombination. ‘Empty node’ refers to an ancestral sequence that is no longer present in the final simulation. Variants 1, 2 and 3 correspond to variants from two different clades in the network. (**E**) Simulated networks were compared to inferred networks based on output antibody sequences. Both correctly and incorrectly inferred networks are visualized. Each node depicts a unique variant, defined by a unique full-length VH–VL nucleotide sequence. The color of each node corresponds to a customizable repertoire feature (e.g. isotype, organ and phenotype) of cells within that variant of a certain isotype. Edges depict clonal relationships. Black nodes correspond to unmutated reference germline.

**Fig. 3.**
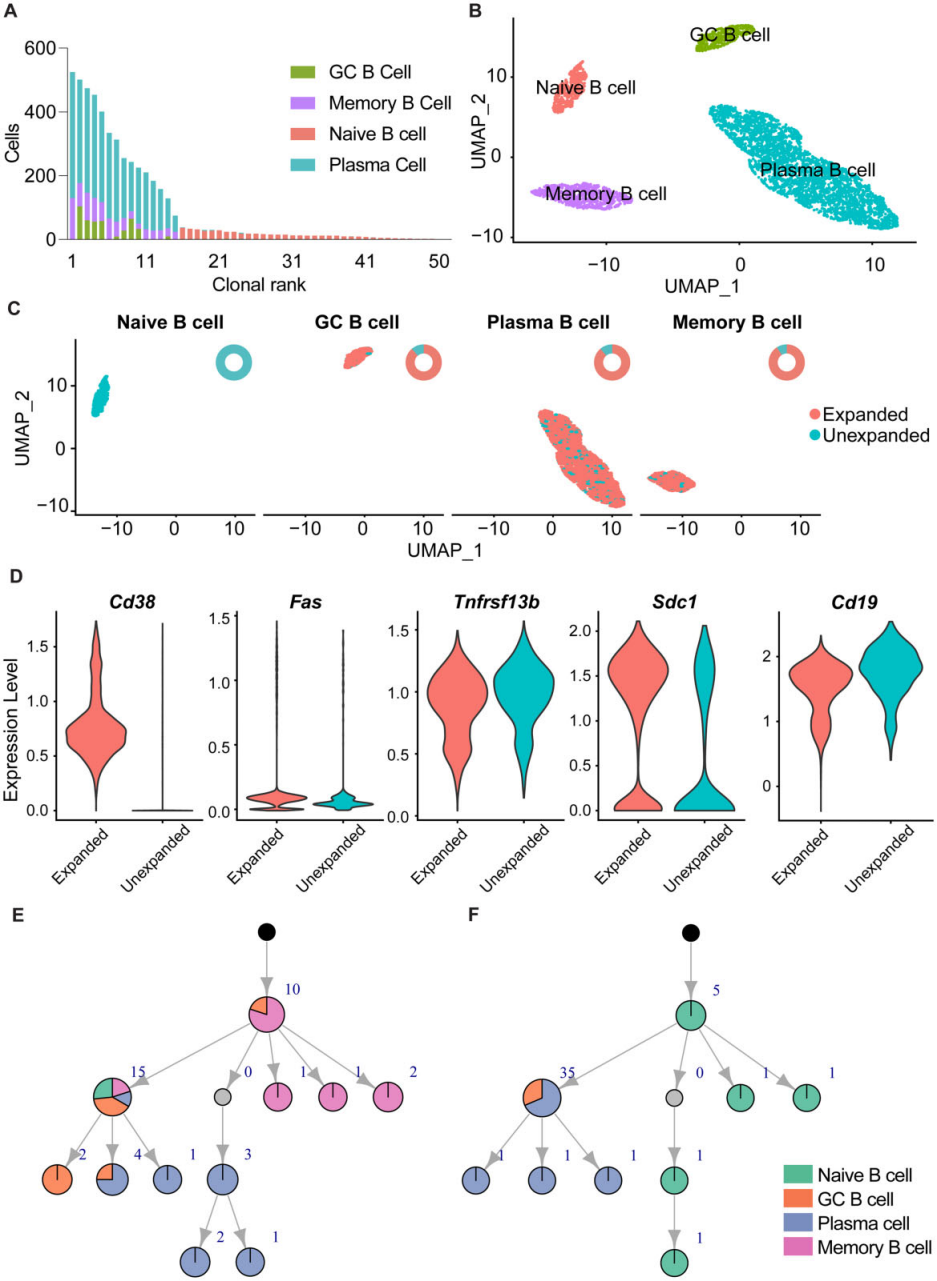
Echidna integrates gene expression and immune receptor during single-cell simulation. (**A**) Preferential expansion of B cell subsets based on cell phenotype. Color corresponds to the number of cells within a given clone of a certain phenotype. Clone is defined as all cells belonging to a single simulated V(D)J recombination event. (**B**) Uniform manifold approximation projection (UMAP) highlighting distinct transcriptional profiles of naive, germinal center (GC), memory and plasma cells. (**C**) UMAP highlighting expanded and unexpanded clones. Expanded clones are defined as those clones supported by two or more unique cells. The donut plots on the up-right corner indicate the proportion of expanded and non-expanded cells in each phenotype. (**D**) Normalized expression of *Cd38,Fas,Taci,Cd138* and *Cd19* separated by expanded versus unexpanded cells. (**E**) Simulated clonal lineage depicting that certain B cell subtypes can be customized to preferentially undergo somatic hypermutation or clonal expansion.

In the case of B cells, Echidna can simulate SHM and clonal evolution in a time-resolved manner. Specifically, at each step, each cell has a probability to undergo BCR diversification using three distinct mutational models. While the simplest method involves a uniform distribution for each nucleotide to mutate to another base, a second option favors transitions relative to transversions. Finally, a third option is to introduce mutations following an experimentally determined 5-mer mutation model, in which neighboring nucleotides influence transition probabilities (Cui *et al.*, 2016; Yaari *et al.*, 2013). At each simulated time step, each cell has a probability to proliferate and incur SHMs, thereby giving rise to either clonally expanded antibody sequences or variants within an individual clone. While it is possible to modulate the method and number of SHM-derived clonal variants, under default parameters, Echidna applies uniform SHM probability to all cells, although the user can preferentially introduce more mutations to class-switched B cells by tuning isotype-specific SHM probability parameters (Fig. 2A). In addition to recovering the full-length antibody sequence associated with each simulated cell barcode, Echidna furthermore provides the mutational network depicting the evolutionary history for each B cell, thereby following the ground truth of B cell evolution (Fig. 2B). The mutational networks contain information pertaining to clonal expansion of individual antibody variants (node size), the fraction of cells with a given isotype (node color) and the clonal relationships (edges). Although both mutations and cell death result in the removal of intermediate variants from the final list of output sequences, an additional mutational network including intermediate nodes with their corresponding sequences can be provided (Fig. 2C). The information from the mutational network and the output sequences can be integrated to investigate how sequence-level mutations dictate clonal relationships (Fig. 2D). To demonstrate Echidna’s ability to benchmark clonal lineage reconstruction algorithms and tools, we simulated 20 B cell networks and supplied the output sequences and respective germlines into a previously utilized pipeline that creates B cell mutational networks based on converting pairwise edit distances to adjacency matrices (Neumeier *et al.*, 2022a). Comparing the topologies of the simulated and inferred networks, we could demonstrate that 19 of the 20 simulated networks were correctly predicted, with the major divergence due to the presence of germline-like B cells which closely resemble the unmutated reference germline (Fig. 3E, Supplementary Fig. S5). We next questioned the extent of similarity between our simulated networks to those inferred from experimental single-cell immune repertoire sequencing data. We therefore simulated murine B cell phylogenetic networks and compared the resulting topologies to the 20 most expanded class-switched B cell networks from murine bone marrow plasma cells isolated following serial protein immunizations (Neumeier *et al.*, 2022a). Networks were compared by their depth, size, breath and imbalance. The imbalance was measured by Sackin Index, which is defined as the sum of the depth of all leaves. This comparison demonstrated that our synthetic networks were able to recapitulate topological features quantifying both evolutionary depth and breadth for specific experimental repertoires (Supplementary Fig. S8).

### 3.4 Integrated simulation of immune receptors and transcriptomes

Echidna can simulate transcriptomes by mimicking the gene expression levels of different cell types based on either user-supplied distributions for each gene or by extracting expression values from user-supplied single-cell or bulk sequencing data. We have additionally provided baseline transcriptional states using publicly available single-cell immune repertoire sequencing data generated by either 10x Genomics or recent publications (Bieberich *et al.*, 2021; Kuhn *et al.*, 2022; Neumeier *et al.*, 2022a). Furthermore, we have included a pipeline to generate gene expression data using VAEs, which could reproduce experimental phenotypes, such as effector, memory and exhausted T cells (Supplementary Fig. S9). The simulated transcriptome data is compatible with widely used scSeq analysis tools, such as Seurat, and can be used to analyze and visualize phenotypic heterogeneity, transcriptional cluster distributions and the expression of individual genes (Supplementary Fig. S3). We next demonstrated the ability to integrate simulated adaptive immune receptors with gene expression information. We first generated BCRs and their corresponding transcriptomes at the single-cell resolution and quantified the cluster membership for the 50 most expanded B cell clones using parameter settings where the selected variants possess higher clonal expansion rates and were also forced to switch to certain cell phenotypes (Fig. 3A and B). The expanded clones with selected variants could be visualized on the UMAP, which revealed that clonally expanded cells were more likely to be in the cluster corresponding to the plasma cell phenotype relative to naive, germinal center (GC), and memory B cells (Fig. 3C). This was further exemplified by quantifying normalized expression values of genes defining each of these subtypes, with plasma cell marker *Cd138* (*Sdc1*) increased relative to markers characteristic of the other B cell subsets (*Cd19 andFas*) (Fig. 3D). In addition to seamlessly integrating simulated adaptive immune receptors into the popular R package Seurat, the transcriptional phenotypes and clusters can additionally be integrated into a mutational model that allows certain antibody variants within a given clone to occupy distinct transcriptional states. To demonstrate this, we simulated two mutational networks, one where the transcriptional probability matrix was constant for all variants; another which had a selected variant preferentially determined to become plasma cells. These plasma cells were given a higher SHM probability over other cell types in the phenotype-specific SHM probability parameter, thus they exhibit more expansion and mutation activities (Fig. 3E), highlighting the ability of Echidna to simulate variant-specific phenotype switching and phenotypic-driven SHM.

### 3.5 Integration of echidna repertoire simulation to spatial transcriptomics gene expression data

**Fig. 4.**
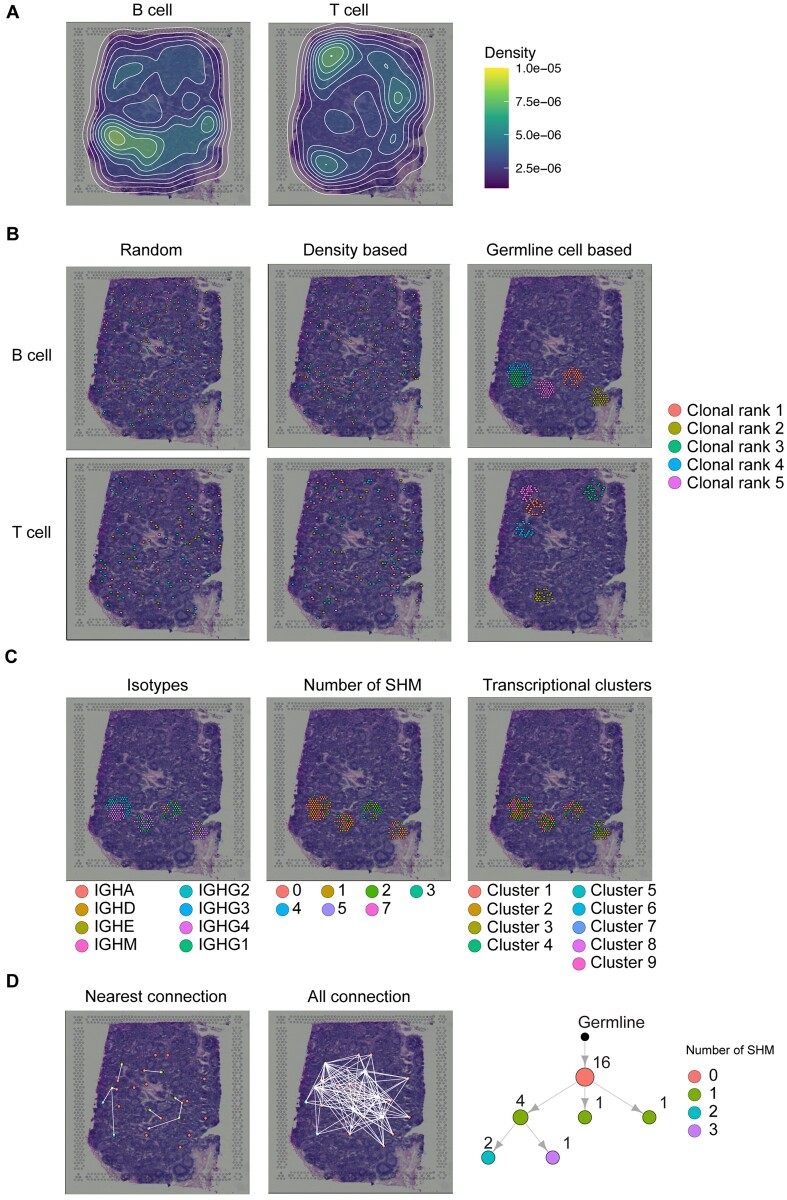
Integration of Echidna simulation with spatial transcriptomics data. (**A**) Density contour plots of T and B cells detected by a list of markers within spatial transcriptomics data. Colors correspond to the calculated density values. (**B**) Assignment of the five most expanded clones simulated by Echidna according to random, density-based and germline-cell-based methods for T and B cells detected in spatial transcriptomics data. Each color represents a unique clonotype. (**C**) Isotypes, somatic hypermutations and clusters representations of the five most expanded clones according to the germline-cell-based assignment method. The different isotypes and clusters as well as the different number of mutations are each distinguished by different colors. (**D**) Cellular evolution trajectory of cells within the most expanded clonotype. Cells with different somatic hypermutations are represented by different colors. Arrows depict the direction of evolution by linking the nearest (left) or all (middle) potential parent cells to the progenitor cell. The mutational network is shown in the right panel

Current methods to integrate spatial location and single-cell transcriptomics have difficulty additionally obtaining full-length immune receptor information for cells at high throughput. Despite ongoing work to generate experimental data sets that integrate cell location, transcriptome and immune receptor sequence, these datasets currently only contain heavy chain information for a few cells (e.g., roughly 27 T cells for an entire image) (Hudson and Sudmeier, 2022). These challenges have thereby prevented the development of computational methods capable of integrating spatial information into clonal relationships. We therefore demonstrated the potential of Echidna to simulate immune receptor sequences for single B and T cells in a spatially resolved manner. To demonstrate this, we utilized publicly available spatial transcriptomics data (10x Genomics) from human lymph node and breast cancer tissue and simulated immune receptors with multiple methods incorporating cell location. We first annotated cells as B and T cells according to the cell surface marker expression (T cells: *Cd3e*, *Cd4* and *Cd8a*; B cells: *Cd19*, *Sdc1*, *Xbp1*). We next visualized the density of the B- and T-cell populations, which demonstrated that B and T cells largely occupied different regions of the lymph node (Fig. 4A). BCR and TCR repertoires were simulated by Echidna according to the number of cells in each population as determined by gene expression profiles. We implemented three different methods to assign the simulated immune receptor sequences to the annotated B or T cells on the spatial image (Fig. 4B): (i) random assignment of the simulated V(D)J to cells detected by spatial gene expression, (ii) density-based assignment, where the simulated BCR or TCR sequences corresponding to the more expanded clone are preferentially assigned to the locations with higher density in the image, yet are not explicitly assigned as the nearest neighbor and (iii) germline assignment, where the simulated germlines are preferentially assigned to locations based on total B or T cell density. However, following germline assignment to the image, the remaining cells within the clonotype are assigned to the nearest unassigned B cells on the image based on the Euclidean distance from the germline. Once all cells of a given clonotype are assigned for methods (ii) and (iii), the density distribution is recalculated excluding the B or T cells on the image that have been assigned. This results in both methods having cells preferentially located relatively close to the germline. The major difference between (ii) and (iii) is that, in the latter, cells within a given clonotype are required to be the closest possible B cell. In contrast, in the former, they follow the same density distribution used to assign a germline to the image. These three methods lead to different cell distributions, as shown in the density contour map of the most expanded clone (Supplementary Fig. S6). As immune receptor sequence and spatial gene expression data are integrated, it was further possible to display immune repertoire and transcriptional features, such as SHM, antibody isotype, transcriptional cluster membership and marker gene expression level for individual clonotypes on the image (Fig. 4C, Supplementary Fig. S7). Furthermore, incorporating SHM data and spatial location provided the possibility to develop bioinformatics pipelines capable of inferring spatially resolved evolutionary trajectories. We mapped simulated phylogenetic networks onto the image and subsequently visualized the directionality of SHMs within the most expanded clonotype (Fig. 4D). Taken together, we could generate a novel data structure that could be used to develop a bioinformatic pipeline to integrate gene expression phenotypes, immune repertoire information and spatial location.

## 4. Discussion

Recent technological advancements in single-cell immune repertoire sequencing have provided insight into how clonal selection relates to cellular phenotypes, thereby paving the way to simultaneously model gene expression with adaptive immune receptor sequences. Here, we developed a simulation framework and further exemplified how simulated repertoires can be leveraged to benchmark and develop bioinformatics software relating to experimental datasets. Although multiple simulation frameworks have been previously developed for immune repertoire simulation (Davidsen and Matsen, 2018; Safonova *et al.*, 2015; Yermanos *et al.*, 2017; Weber *et al.*, 2020), it was not possible to link these repertoire features at the single-cell level. There remains a lack of software specifically tailored to generating adaptive immune receptors at the single-cell resolution and with corresponding transcriptome information. Echidna bridges the gap between single-cell transcriptome and immune repertoire sequencing, providing information relevant to both fields at the single-cell resolution. An additional advantage of Echidna is that mutations are introduced at the clonal level in a time-resolved manner while also providing the true, known network structure that generated a given clonal lineage. This information can be used to explore how various parameters dictating the inference of phylogenetic trees and similarity networks relate to either the immune repertoire or gene expression phenotypes (DeWitt *et al.*, 2018). Although we have provided default parameter values derived from experimental sequencing data from 10x Genomics and recently published data (Bieberich *et al.*, 2021; Kuhn *et al.*, 2022; Neumeier *et al.*, 2022a), Echidna similarly allows the user to customize each feature of the simulation, including the initial clonal diversity generated via V(D)J recombination, clonal expansion, transcriptional states, and their respective transition probabilities, and, for B cells, both SHM and isotype distributions.

This customizability further allows preexisting simulation frameworks to generate initial expression or repertoire data that can be supplied as input to Echidna. Thus, the user can first simulate V(D)J repertoires using existing tools that integrate recombination statistics from real sequencing reads [e.g. IGOR (Marcou *et al.*, 2018)], incorporate CDR3 amino acid probability distributions [e.g. OLGA (Sethna *et al.*, 2019)], model pre- and post-repertoire selection [e.g. SoN(N)ia (Isacchini *et al.*, 2021; Sethna *et al.*, 2020)], or leverage VAEs to fit TCR sequence distributions (Davidsen *et al.*, 2019). Following external simulation, immune receptor sequences can be supplied as input to Echidna, which can then add additional features such as time-resolution, single-cell resolution, transcriptional phenotypes and spatial location. While we have demonstrated that our two methods of V(D)J recombination perform similarly compared to randomly generated antibody sequences, it is likely that further optimization or use of external tools can further decrease the divergence between experimental and synthetic repertoires. A similar external simulation can be performed for gene expression data, which can be supplied as input for the initial transcriptional distributions. Thus, tools such as Polyester (Frazee *et al.*, 2015) and Splatter (Zappia *et al.*, 2017), which either simulate sequencing reads or allow parameter estimation from experimental data, can generate the input expression vectors that can subsequently be supplied as input to Echidna. In addition to the potential to integrate external tools, the user can also simulate immune repertoire or gene expression data by supplying experimental repertoire datasets to Echidna. Thus, by supplying data from distinct experimental sources, the user can model features such as batch and donor effects that are inherently present in the underlying expression matrices and starting repertoires. In the current version, Echidna allows users to simulate mutational network features, such as depth, size of the network by tuning the simulation parameters. However, further parameter optimization and exploration and be performed, potentially with methods involving approximate Bayesian computation (Beaumont, 2019).

Modeling immunological processes requires significant approximations and assumptions that may not accurately reflect reality. Congruent with this, our simulation tool relies upon many assumptions at each step in the algorithm. As an example, the default simulation utilizes a simple concatenation method to simulate V(D)J recombination that relies upon selecting germline genes and inserting/deleting nucleotides based on subjective probability distributions. While this produces extremely diverse repertoires, it ignores many of the complexities not immediately observable underlying actual repertoire diversity, hence it lacks a comprehensive data-driven simulation basis (Greiff *et al.*, 2017; Marcou *et al.*, 2018; Slabodkin *et al.*, 2021). We, therefore, have introduced the ability for more sophisticated repertoire simulations involving VAEs (Davidsen *et al.*, 2019; Friedensohn *et al.*, 2020), which, under default parameters, are trained on publicly available data of naive B and T cell repertoires and serve as input for the started repertoire pool. Importantly, we have additionally included the option for users to supply their own repertoires as training data to the VAE, thereby enabling the simulation of a repertoire resembling specialized experimental conditions when post-selection repertoires are desired. While VAEs have been successful in maintaining patterns detected in experimental transcriptomes (Way and Greene, 2018), it remains challenging to measure the divergence between synthetic and experimental data. This is both due to the novelty and cost of current iterations of single-cell immune repertoire sequencing datasets, in addition to the lack of a unified database linking immune receptors to transcriptome. While we showed that synthetic repertoires exhibit comparable features pertaining to transcriptional heterogeneity, B cell evolution, and clonal expansion, significant variabilities between experimental conditions have been reported for these parameters (Attaf *et al.*, 2021; Khatun *et al.*, 2021; Kuhn *et al.*, 2022; Shlesinger *et al.*, 2022). Similarly, a complete model of clonal selection integrating features such as clonal expansion, affinity, and GC reactions has not yet been realized. We have attempted to allow flexibility for these parameters by providing expansion and mutation rates at the level of individual cells and variants, our framework is unlikely to reproduce complicated selection processes such as clonal bursts and GC collapse (De Silva and Klein 2015; Mesin *et al.*, 2016; Victora and Mesin, 2014). As more datasets linking repertoire features (e.g. gene expression, receptor sequence) to functional properties (e.g. affinity, specificity) become publicly available, we can continuously update the generative model underlying our framework, thereby bringing our simulations closer to natural immune repertoires.

Spatial transcriptomics data provides the relationship between transcriptional status of cells and their relative locations within tissue. This powerful tool has been applied across various biological conditions and has led to new discoveries in pathology and disease development in various organs (Baccin *et al.*, 2020; Crosse *et al.*, 2020; Delile *et al.*, 2019; Roth *et al.*, 2020). However, immune receptor sequencing data is not yet integrated into spatial transcriptomics pipelines but will likely be a valuable addition to next-generation spatial transcriptomic experiments. The combination of immune repertoire features and gene expression profiles of specific immune cell populations will improve our understanding of the biological processes in a vast range of contexts, such as the movement and selection of individual B and T cells during germinal center reactions. Echidna’s ability to simulate repertoires onto images thereby will facilitate the development of relevant bioinformatic tools that can be applied to investigate both aspects of phenotypic behavior and clonal evolution on a spatial landscape.

**Data visualization:** Circos plots were created using the function VDJ_circos in Platypus (v3.1), which relies upon the R package circlize (v0.4.13) (Gu *et al.*, 2014). Mutational networks were created using igraph (v1.2) (Csardi *et al.*, 2006). Sequence alignment was performed in Geneious Prime (v.2020.0.3) under default parameters. UMAP, violin plots, and feature plots were performed using Seurat (v4.0.1) (Satija *et al.*, 2015). Plots regarding to spatial transcriptomic data were made using the pipeline provided by 10× based on R Package ggplot2 (v3.3.5) (Wickham, 2016). Remaining plots were produced in Prism Graphpad (v9).

## Supplementary Material

vbac062_Supplementary_Data
