# Supplementary material for: Echidna: integrated simulations of single-cell immune receptor repertoires and transcriptomes
Source: Bioinform Adv. 2022 Sep 2;2(1):vbac062. doi: 10.1093/bioadv/vbac062 (PMC9710610; doi:10.1093/bioadv/vbac062)
Supplement: vbac062_Supplementary_Data [file vbac062_supplementary_data.zip › Supporting_Material_S4.pdf]

Supporting information for:

Echidna: integrated simulations of single-cell immune receptor repertoires and transcriptomes

Jiami Han[1], Raphael Kuhn[1], Chrysa Papadopoulou[1], Andreas Agrafiotis[1], Victor Kreiner[1], Danielle Shlesinger[2], Raphael Dizerens[1], Kai-Lin Hong[1], Cédric Weber[1], Victor Greiff[4], Annette Oxenius[2,], Sai T. Reddy[1], Alexander Yermanos[1,2,3,*]

[1]Department of Biosystems Science and Engineering, ETH Zurich, Basel, Switzerland
[2]Institute of Microbiology, ETH Zurich, Zurich, Switzerland
[3]Department of Pathology and Immunology, University of Geneva, Geneva, Switzerland
[4]Department of Immunology, University of Oslo, Norway
*Correspondence: ayermanos@gmail.com

Methods

Simulating adaptive immune repertoires at single-cell resolution

Relevant simulation parameters for all figures can be found as supplementary information (Table S1) and accompanying code and compiled package can be found at github.com/alexyermanos/echidna. The VDJ recombination simulation portion of the algorithm is adapted from AbSim (Yermanos et al. 2017). Here, we simulate the heavy chain and light chain sequence of an individual cell simultaneously and integrate the relevant sequence and annotation information. For heavy chain simulations, D and J gene segments are first combined, followed by V segments joining to the previously formed D-J segment. In the case of light chains, only V and J segments are joined. Insertions and deletions can occur at the junction site for each fusion event. In the case of insertions, each of the four nucleotides had an equal probability of being selected. The number of inserted nucleotides can range from 0 to 10 under default parameters but can either follow a uniform or a custom probability distribution supplied from an experimental human or mouse-specific model, as specified by the user. For junctional deletions, the sampled number of deleted nucleotides at the end of each germline segment can be customized. However, for all simulations presented here, a range of 0 to 5 nucleotides was selected with a uniform distribution. The V, D, and J segments were sampled using a uniform distribution from all human and mouse germline genes from IMGT (Lefranc et al. 2003), unless otherwise specified. The user can modify their frequency by adding, replicating, or deleting genes in the reference germline genes. For the example presented in this manuscript, IGHV1-11 and IGKV1-131 genes were replicated in the input germline gene list to obtain preferential heavy and light chain gene usage.

The random insertion and deletion processes in the VDJ recombination mechanism might introduce frameshift mutations and generate non-productive sequences before any evolution commences. We have therefore provided an option for limiting the initial repertoire to only productive sequences as determined by using the align and exportAlignments commands from MiXCR (v3.0.1) under default parameters (Bolotin et al. 2015) or using VAE generated productive sequences (section below), and this can be controlled by a single productive vdj.productive. After restricting the initial VDJ recombination events to productive sequences, the repertoire will only use these sequences to initialize new clones. The productive sequences were prepared by first simulating more than 100,000 VDJ sequences for each chain type and species and subsequently filtering out non-productive sequences with MiXCR (Bolotin et al. 2015) from simulated sequences. The productive sequences were additionally annotated with their CDR3 nucleotide and amino acid sequences.

Simulating with variational autoencoders

As an additional option to simulate productive adaptive immune receptors, we employed variational autoencoders (VAEs) to generate new sequences with patterns resembling experimental data (Friedensohn et al. 2020; Davidsen et al. 2019; Eguchi et al. 2020) using R package Keras (JJ Allaire and François Chollet 2020). Our VAE pipeline for immune

receptor sequence simulation is based on the default pipeline from the variational_autoencoder function of the Keras package. VAE models were trained based on publicly available repertoire sequencing data (Table S2). Although Echidna does not alter the VAE generation algorithm, we have added data pre-processing functions to ensure adaptability to immune repertoire sequencing data. Input data sets were first one-hot encoded and subsequently split into two evenly sized folds. One-fold was used for validation and the remaining folds were used for training, where the maximum range of latent points was then recorded after model training. For each chain type, 1,000,000 points were drawn within the recorded range in latent space containing the training data (Figure S3). The predictive values of decoded sample points were then reconstructed into nucleotide sequences based on the highest predicted value of the VAE model. A threshold of the four predicted values for the four nucleotide bases was set to determine the end of the sequence. The base was considered to be null if the predicted value was lower than the threshold (0.05). Final sequences were generated from the generator model using the predict function from stats R package with the generative object supplied as input under default parameters. The final VDJ sequences were then filtered by MiXCR to ensure only productive sequences as previously described, which were embedded in the starting repertoire sequence pool for subsequent simulation.

To quantify the Kullback-Liebler divergence (KLD) of probability distribution between Experimental training data and sequences generated from VAE model trained by the experimental training data, we took 1000 mouse BCR heavy chain sequences from 10x Genomics database (link: https://www.10xgenomics.com/resources/datasets/pbm-cs-from-c-57-bl-6-mice-ig-enrichment-from-amplified-c-dna-1-standard-3-0-0) for VAE model training. Another 1000 BCR heavy chain sequences were simulated with Echidna naïve VDJ appending method as the baseline reference of natural mouse BCR sequences under default parameters. To ensure that the length of each group of sequences is consistent for later KLD comparison, experimental sequences and Echidna appended sequences were aligned, and the alignment result of experimental data were used as the training data so that the length of VAE generated sequences remained the same length as the alignment result of experimental sequences and appended sequences. The trained VAE model generated 1000 sequences. Furthermore, 1000 randomly synthesized sequences were made as a random reference, where each position in the sequence has equal probability to be any nucleotide base or alignment gap, and the length of the sequences are all same as the above-mentioned alignment result. Position-wise base frequencies were calculated for each group of sequences, then KLD was computed between 1) training data supplied as input to the VAE and the VAE-generated sequences, 2) training data supplied as input to the VAE and the naïve VDJ recombination simulation method Echidna involving appending germline genes and randomly inserting or deleting nucleotides at the junctions (termed Appended) 3) training data supplied as input to the VAE and nucleotide sequences generated with each base having an equal probability of each base (A, T, G, C), 4) VAE-generated sequences and randomly-generated nucleotide sequences, and 5) VAE-generated sequences and Echidna's Appended method. The KLD was calculating using the function from

LaplacesDemon package in R under default parameters (“Lernen im Netz - bayesian-inference.com” n.d.).

Somatic Hypermutation

The method to simulate SHM for B cells is adapted from AbSim (Yermanos et al. 2017), which offers multiple methods to introduce sequence diversity. First, a “Poisson'' method can mutate each nucleotide randomly at a user-defined probability at every position in the immune receptor. Second, the “Data-driven” method involves targeted mutations in inferred CDR locations, in addition to allowing different mutation rates for nucleotide transitions or transversions. Third, the “motif” method considers the influence of neighboring bases and detects 5-mer sequence motifs in the sequence and applies nucleotide-specific substitution probabilities to nucleotides located in the middle of 5-mer motifs. The transition probabilities were determined from previous studies modeling mutational frequencies across multiple antibody datasets (Yaari et al. 2013). If desired, the user can additionally update the 5-mer mutational frequencies with custom distributions. Additionally, a “wrc” method is available, where the WRC motifs in the antibody sequence will preferentially undergo somatic hypermutation substitution based on the relevant 5-mer motifs (Yaari et al. 2013). A combination of any of the above-mentioned methods with user-defined weights is possible. Finally, the user can specify custom SHM probabilities for certain repertoire features, such as transcriptional phenotypes and antibody isotypes.

Simulating gene expression

The gene expression level of a certain defined phenotype can be represented with a numeric base vector, where each element corresponds to the normalized expression level for each gene. The final expression level for an individual cell will be sampled based on the base vector in combination with a user-defined noise parameter. The parameter for noise distribution can be estimated by supplying experimental datasets to Echidna and can be distinct for each specified transcriptional phenotype. Therefore, cells of the same phenotype will sample from an identical base vector, but their actual value of expression level will fluctuate with the introduction of noise. Finally, gene expression vectors will be combined into a gene expression matrix that is compatible with common RNA-seq analysis frameworks, such as Seurat (Satija et al. 2015). For B cells, the user can choose any combination of the four phenotype switching strategies by enabling or disabling any of them: (i) SHM dependent: If SHM occurs to a cell, it will switch to one of the user-supplied possible phenotypes. If there is more than one potential phenotype, the probability to change from one phenotype to the next is determined by the transition matrix. In the default transition matrix, the chance of GC B cells switching to plasma cells and memory B cells are 2/3 and 1/3, respectively. Whereas, under default parameters, plasma cells would not transition to other phenotypes when undergoing SHM, as their transition probabilities to the other cell types are set to 0. (ii) Class switching dependency: Similarly, phenotype switching can occur once a cell undergoes class-switch recombination, again defined by a transition matrix containing the probabilities to change from one state to another state. (iii) Variant selection

dependency: within each clone, when a new variant (or node in the network) is generated, it has a chance to be selected and possess a different expansion rate. Cells belonging to the selected variants will skip naïve phenotype state, and become one of the other cell types according to the proportion of probability in phenotype transition matrix. Therefore, certain B cell sequences (e.g., certain affinities) can be modeled to certain phenotypes based on antibody sequence. (iv) Phenotypes switch randomly following either a default transition matrix or user-supplied transition matrix.

To generate default gene expression vectors, we incorporated data from either 10x Genomics or previous single-cell immune repertoire sequencing studies containing either human or mouse B and T cells (Table S3) (Bieberich et al. 2021; Neumeier et al. 2022; Kuhn et al. 2022). The data was prepared by first pooling all cells (separately for humans and mice) and subsequently sorting cells of desired phenotypic markers. The average gene expression levels across all cells matching the phenotypic marker definitions were then extracted as base vectors for each phenotype (Table S3). While these serve as default parameters, the package is intended to receive custom expression vectors based on a user's individual dataset or interest. The VAE-based method for transcriptome simulation was performed using the R package VAExprs (Jung 2021) using 5,007 features from 10,000, randomly selected, virus-specific CD8 T cells (Kuhn et al. 2022). The features were selected by performing a variable feature analysis with the function FindVariableFeatures from Seurat package and 5,000 most variable markers were selected in addition to a chosen list of CD8 T cell-specific markers: BCL6, STAT3, STAT4, KLF4, SMAD1, CD3E and CD8A. The training VAE model was set up with a batch size of 50, an intermediate dimension of 256, an epoch size of 100, and a latent dimension of 100. The whole matrix of 5,007 features and 10,000 cells was used as training dataset. VAE-based transcriptome simulation can be used as a complementary method, and the output data can be integrated with immune receptor sequences from our tool.

Inferring mutational networks

Mutational networks were inferred as previously described (Neumeier et al. 2022), which utilizes the R package igraph (Csardi, Nepusz, and Others 2006). First, the pairwise edit distance was calculated for each appended, full-length heavy and light chain sequence (including the reference germline), thereby generating a distance matrix for each clone using the stringdist R package (van der Loo 2014). This distance matrix was then used to determine the order in which sequences were added to the network. The unmutated germline reference gene initializes each network, and then the sequence with the smallest distance was added to the most similar sequence in the network in an iterative manner. Edit distance ties were resolved by randomly selecting from the potential nodes within the network. The reference germline sequence and appended heavy and light sequence of each clone are available in the simulation output.

Simulating immune receptor sequences for spatial transcriptomic data

Spatial transcriptomics data of the human lymph node were provided as public data from 10X Genomics under their datasets section on their website. B and T cells were annotated based on marker gene expression (*CD19*, *SDC1*, *XBP1* for B cells and *CD3E*, *CD4* and *CD8A* for T cells). Equivalent numbers of BCR and TCR sequences were simulated with Echidna. The simulated BCR and TCR sequences can be assigned to cells in spatial data by three strategies: (i) BCR or TCR sequences were randomly assigned to cells in spatial transcriptomic data. (ii) Density-based method: the cell density on the image was calculated by the function approxfun in the stats package (v3.6.2), which returns the approximated density value at the coordinate of each cell. Cells in spatial transcriptomics data were ranked by decreasing cell density, whereas the simulated BCR or TCR sequences were ranked by decreasing size of the clone. After sorting the two counterparts in the desired order, they were combined by rows such that the cells from the higher density part of the image will get a clonal identity from a more expanded clone. This sequence assignment process was iterated for each clone, and before the next round of assignment starts, cell density is recalculated after excluding cells that obtained their BCR or TCR sequences during the last iteration. This thereby maintains the density within every clone independently from other cells involved in the previous sequence assignment step and ensures that highly expanded clones have higher cell densities. (iii) The calculation of the density for each cell is the same as method (ii). The sequence assignment process occurs repeatedly for each simulated clone, where the first generated sequence in the clone, namely the germline sequence, will be assigned to the cell at the location with the highest cell density, and the rest of the sequences in the clone will be assigned to cells close to the germline cell. The Euclidean distance of cells to the germline cell was calculated based on the cell coordinates by applying the Pythagorean theorem. The distance is calculated as follows: $z = \sqrt{x^2 + y^2}$, with $x = |x_{germline} - x_{cell}|$ and $y = |y_{germline} - y_{cell}|$). As with the second method, cell density will be updated between each assignment process, to keep cell density within every clone independent from previous sequence assignment events.

Spatial transcriptomics and immune receptor data analysis

Isotype, SHM, and transcriptional cluster information were integrated into the spatial image by matching immune receptors with existing cell barcodes on the experimentally-derived image. The total numbers of somatic hypermutations between the sequences and the germline sequence of each clone were calculated using the function get.seq.distance from Echidna. Cluster identity of the cell populations was obtained by performing unsupervised clustering with Seurat package but can also be alternatively defined by the user. Cells from the five most expanded clones were plotted as dots in different colors according to their isotype, number of SHMs and cluster identities, and overlaid on the spatial image. Spatial cellular evolution trajectories were visualized by mapping the mutational network of a clone onto the spatial image. Cells with identical BCRs were organized into identical nodes in the mutational network. Arrows were drawn on the image between cells with ancestor-progenitor relationships based on the simulated clonal evolution. The first set of arrows indicate those nodes that were closest to germline, as the hypothetical germline sequence

was not assigned onto any cells in the image. Arrows were drawn either between all possible cells for each ancestor-progenitor relationship or only between the nearest ancestor-progenitor cells within a node.

References

Bieberich, Florian, Rodrigo Vazquez-Lombardi, Alexander Yermanos, Roy A. Ehling, Derek M. Mason, Bastian Wagner, Edo Kapetanovic, et al. 2021. "A Single-Cell Atlas of Lymphocyte Adaptive Immune Repertoires and Transcriptomes Reveals Age-Related Differences in Convalescent COVID-19 Patients." *Frontiers in Immunology* 12: 2737.

Bolotin, Dmitriy A., Stanislav Poslavsky, Igor Mitrophanov, Mikhail Shugay, Ilgar Z. Mamedov, Ekaterina V. Putintseva, and Dmitriy M. Chudakov. 2015. "MiXCR: Software for Comprehensive Adaptive Immunity Profiling." *Nature Methods* 12 (5): 380–81.

Csardi, Gabor, Tamas Nepusz, and Others. 2006. "The Igraph Software Package for Complex Network Research." *InterJournal, Complex Systems* 1695 (5): 1–9.

Davidsen, Kristian, Branden J. Olson, William S. DeWitt 3rd, Jean Feng, Elias Harkins, Philip Bradley, and Frederick A. Matsen 4th. 2019. "Deep Generative Models for T Cell Receptor Protein Sequences." *eLife* 8 (September). https://doi.org/10.7554/eLife.46935.

Eguchi, Raphael R., Namrata Anand, Christian A. Choe, and Po-Ssu Huang. 2020. "IG-VAE: Generative Modeling of Immunoglobulin Proteins by Direct 3D Coordinate Generation." *bioRxiv*. https://doi.org/10.1101/2020.08.07.242347.

Friedensohn, Simon, Daniel Neumeier, Tarik A. Khan, Lucia Csepregi, Cristina Parola, Arthur R. Gorter de Vries, Lena Erlach, Derek M. Mason, and Sai T. Reddy. 2020. "Convergent Selection in Antibody Repertoires Is Revealed by Deep Learning." *bioRxiv*. https://doi.org/10.1101/2020.02.25.965673.

Jung, Dongmin. 2021. "Generating Samples of Gene Expression Data with Variational Autoencoders." December 15, 2021. https://bioconductor.riken.jp/packages/3.15/bioc/vignettes/VAExprs/inst/doc/VAExprs.html.

Kuhn, Raphael, Ioana Sandu, Andreas Agrafiotis, Kai-Lin Hong, Danielle Shlesinger, Daniel Neimeier, Doron Merkler, Annette Oxenius, Sai T. Reddy, and Alexander Yermanos. 2022. "Clonally Expanded Virus-Specific CD8 T Cells Acquire Diverse Transcriptional Phenotypes During Acute, Chronic, and Latent Infections." *Frontiers in Immunology* 13 (February): 782441.

Lefranc, Marie-Paule, Christelle Pommié, Manuel Ruiz, Véronique Giudicelli, Elodie Foulquier, Lisa Truong, Valérie Thouvenin-Contet, and Gérard Lefranc. 2003. "IMGT Unique Numbering for Immunoglobulin and T Cell Receptor Variable Domains and Ig Superfamily V-like Domains." *Developmental and Comparative Immunology* 27 (1): 55–77.

"Lernen im Netz - bayesian-inference.com." n.d. Accessed July 30, 2022. http://www.bayesian-inference.com/software.

Loo, M. P. J. van der. 2014. "The Stringdist Package for Approximate String Matching. R J. 6, 111--122." *Molecular Therapy: Methods & Clinical Development*.

Neumeier, Daniel, Alessandro Pedrioli, Alessandro Genovese, Ioana Sandu, Roy Ehling, Kai-Lin Hong, Chrysa Papadopoulou, et al. 2022. "Profiling the Specificity of Clonally Expanded Plasma Cells during Chronic Viral Infection by Single-Cell Analysis." *European Journal of Immunology* 52 (2): 297–311.

Satija, Rahul, Jeffrey A. Farrell, David Gennert, Alexander F. Schier, and Aviv Regev. 2015. "Spatial Reconstruction of Single-Cell Gene Expression Data." *Nature Biotechnology* 33 (5): 495–502.

Yaari, Gur, Jason A. Vander Heiden, Mohamed Uduman, Daniel Gadala-Maria, Namita Gupta, Joel N. H. Stern, Kevin C. O'Connor, et al. 2013. "Models of Somatic Hypermutation Targeting and Substitution Based on Synonymous Mutations from High-Throughput Immunoglobulin Sequencing Data." *Frontiers in Immunology* 4 (November): 358.

Yermanos, Alexander, Victor Greiff, Nike Julia Krautler, Ulrike Menzel, Andreas Dounas, Enkelejda Miho, Annette Oxenius, Tanja Stadler, and Sai T. Reddy. 2017. "Comparison of Methods for Phylogenetic B-Cell Lineage Inference Using Time-Resolved Antibody Repertoire Simulations (AbSim)." *Bioinformatics* 33 (24): 3938–46.

A

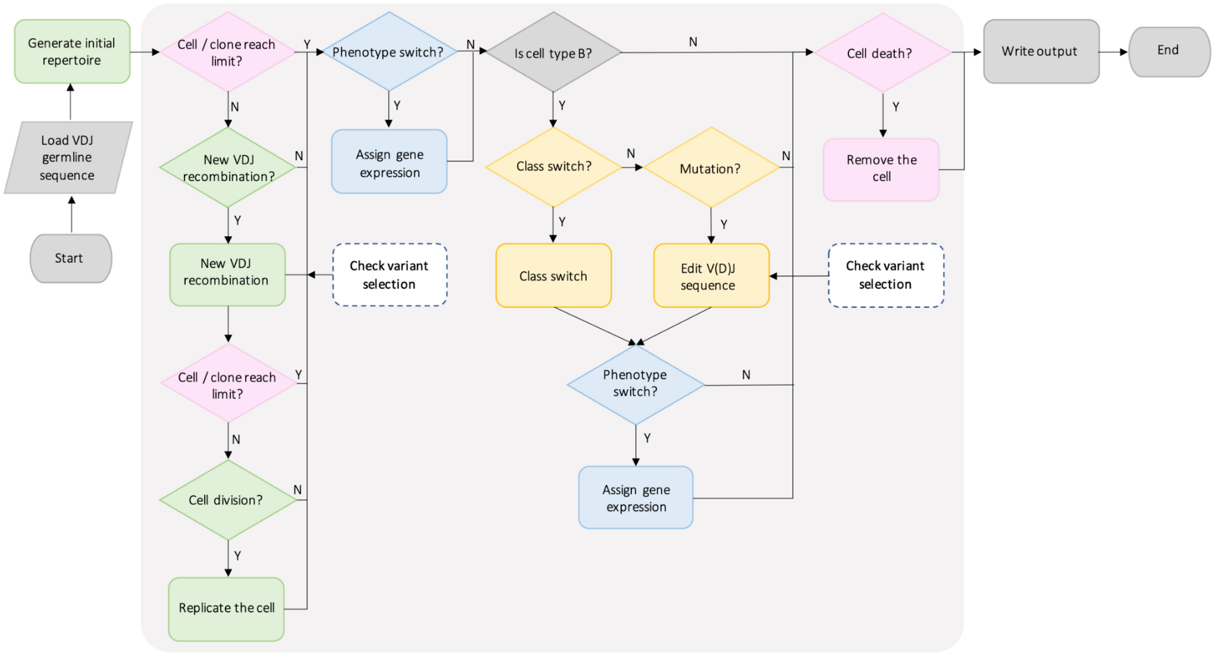

B

10x Genomics

barcode	contig_id	cdr3_aa	chain	v_gene	d_gene	j_gene	c_gene
AAACCTGTCCATTCTA-1	AAACCTGTCCATTCTA-1_contig_1	CATYYYGSSYPFAYW	IGH	IGHV5-17	IGHD1-1	IGHJ3	IGHM
AAACCTGTCCATTCTA-1	AAACCTGTCCATTCTA-1_contig_2	CFQGSHVPPTF	IGK	IGKV1-117	None	IGKJ1	IGKC
AAACCTGAGGAACTGC-1	AAACCTGAGGAACTGC-1_contig_1	CARKGLYYGSSYRTT	IGH	IGHV3-6	IGHD1-1	IGHJ2	IGHD
AAACCTGAGGAACTGC-1	AAACCTGAGGAACTGC-1_contig_2	CQQYYSYPLTF	IGK	IGKV8-30	None	IGKJ5	IGKC

Simulated data

barcode	contig_id	cdr3_aa	chain	v_gene	d_gene	j_gene	c_gene
ACGTTAATACCCATAT-1	ACGTTAATACCCATAT-1_contig_1	CATTMVTYWYFDVW	IGH	IGHV1-12	IGHD2-7	IGHJ1	IGHM
ACGTTAATACCCATAT-1	ACGTTAATACCCATAT-1_contig_2	CGRILRQLGLFAYW	IGK	IGKV1-131	None	IGKJ2	IGKC
TGTGATTAGCGCGCAG-1	TGTGATTAGCGCGCAG-1_contig_1	CGREYLRNSWFAYW	IGH	IGHV1-11	IGHD5-2	IGHJ3	IGHD
TGTGATTAGCGCGCAG-1	TGTGATTAGCGCGCAG-1_contig_2	CAFITTATPGSWFAYW	IGK	IGKV17-127	None	IGKJ2	IGKC

FIgure S1. A. Computational workflow of single-cell immune repertoire and transcriptome simulations using Echidna. Steps in grey frame are applied to each cell in the repertoire in each loop. B. Simulated repertoire output mirrors 10x Genomics' cellranger output from experimental sequencing datasets.

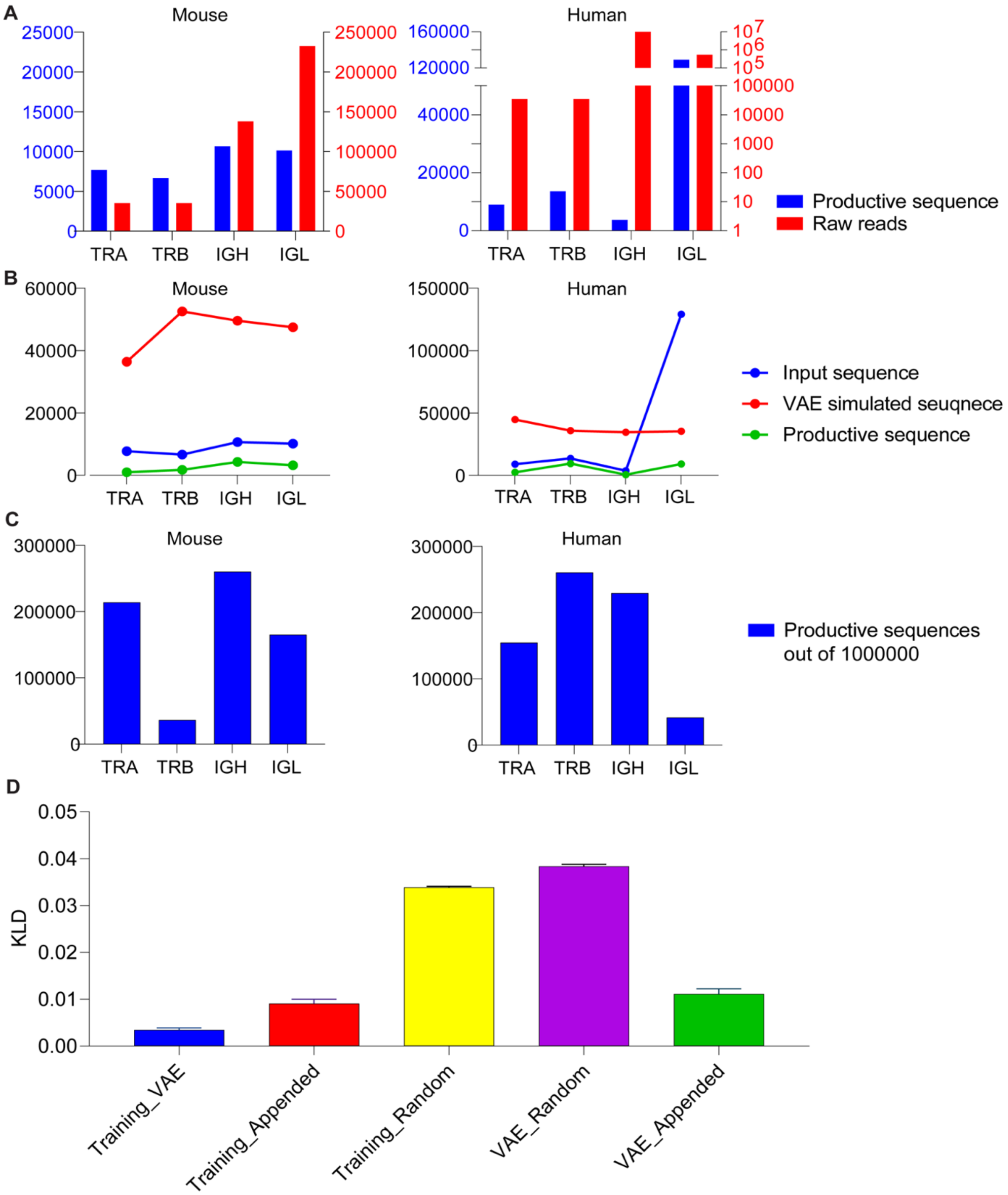

Figure S2. Fraction of productive V(D)J recombination events. A. The number of raw reads and the number of productive adaptive immune receptor sequences per variable chain for human and mice from publicly available data. B. The number of productive input sequences used as input to train the variational autoencoder (VAE), the number of simulated sequences by the VAE generative model, and the number of productive simulated sequences as determined by MiXCR. C. The number of productive sequences out of 1,000,000 simulated VDJ recombination events using a naive model of appended reference alleles from IMGT together with uniform probabilities of insertions and deletions at each simulated junction. D. Kullback-Leibler divergence of nucleotide base distribution along murine B cell VDJ sequences either synthetically or experimentally generated. Comparisons (from left to right) are as follows 1. Experiment (Training) BCRs versus VAE-generated sequences, 2. VAE-generated sequences versus Echidna's naïve VDJ recombination (appending germline genes and random insertion/deletion at CDR3), 3. Experiment (Training) BCRs versus randomly generated BCR sequences (each nucleotide position has a random chance for any of the four bases), 4. VAE-generated sequences versus randomly generated BCR sequences (each nucleotide position has a random chance for any of the four bases), and 5. VAE-generated sequences versus Echidna's naïve VDJ recombination (appending germline genes and random insertion/deletion at CDR3). Error bars indicate the standard error of mean.

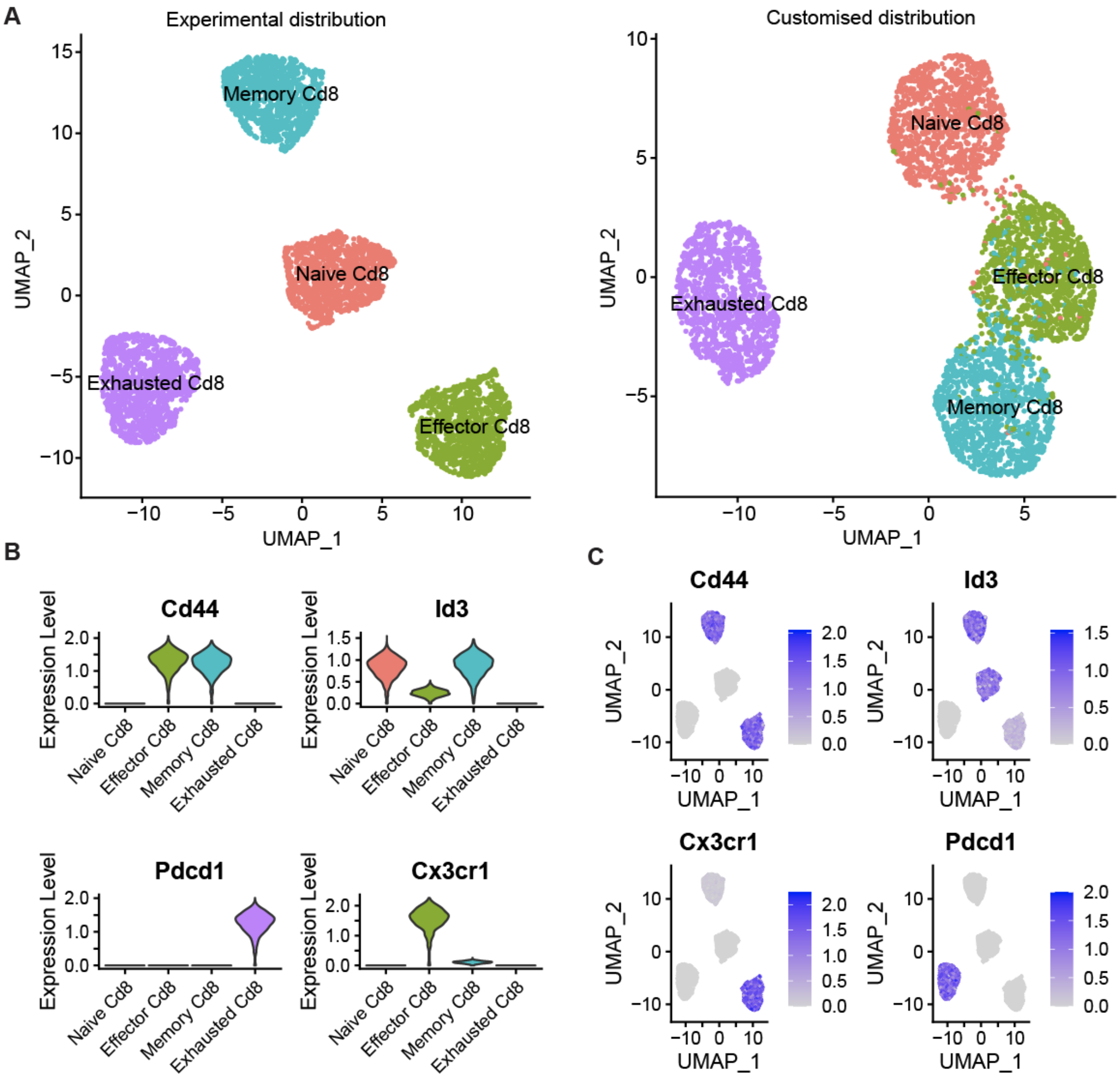

Figure S3. Echidna simulates transcriptomes based on experimental sequencing data. A. Uniform manifold approximation projection (UMAP) depicting transcriptional landscape of simulated CD8 T cell repertoires based on phenotypes from either experimental data or customized gene expression distributions. Color corresponds to clusters as determined by unsupervised clustering. Simulation output was used as input to the common scSeq tool Seurat. Each point represents a cell. Left panel shows the simulation with distinct cell population clusters, right panel shows the cluster with partially overlapped clusters. B–C. Normalized expression of simulated T cells for select phenotype-defining genes visualized by either volcano plots or UMAP.

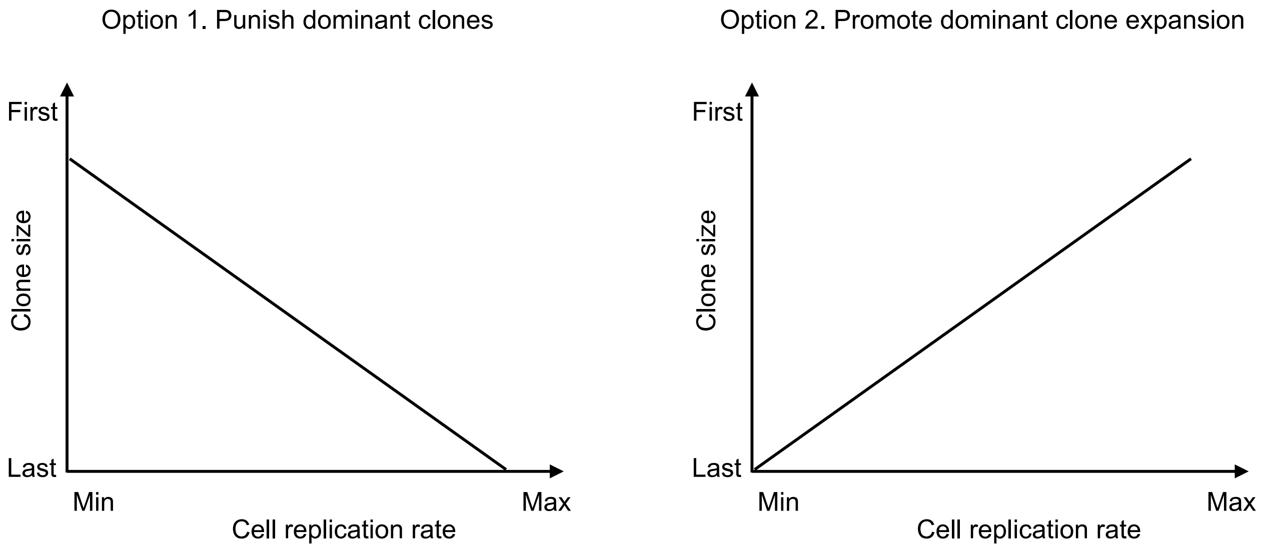

Figure S4. Two Options for cell replication rate adjustment. Option 1: Punish clones with more cells by assigning relatively low cell replication rate in the defined range. Option 2: Promote growth of dominant clones by assigning cell replication rate proportionally to the current number of cells in the clone to which it belongs.

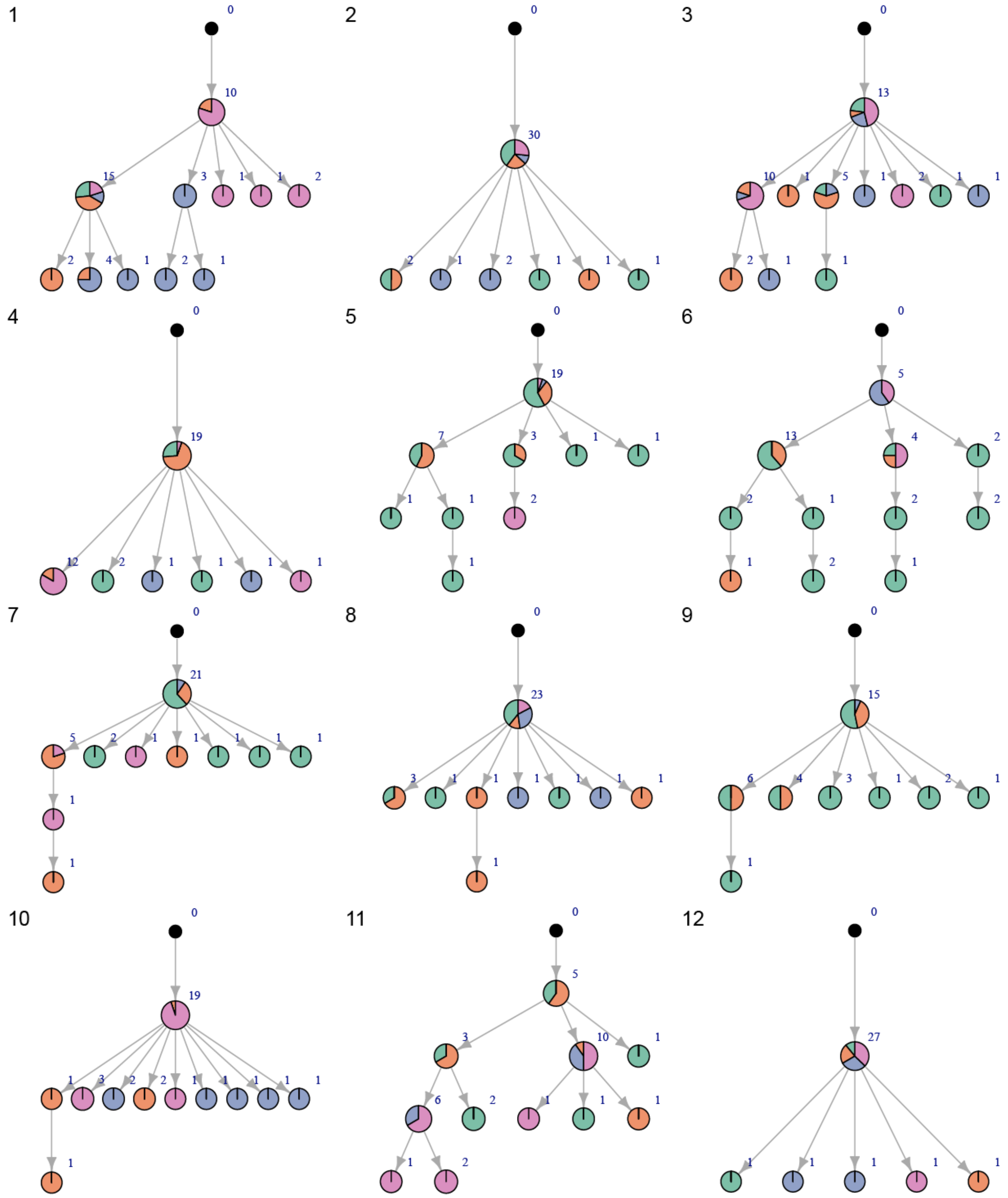

Figure S5. Examples of correctly inferred networks based on output sequences from Echidna. Color corresponds to user-tunable cell phenotype parameters. Nodes correspond to unique, full-length nucleotide variants. Node label corresponds to the number of cells with an identical antibody sequence.

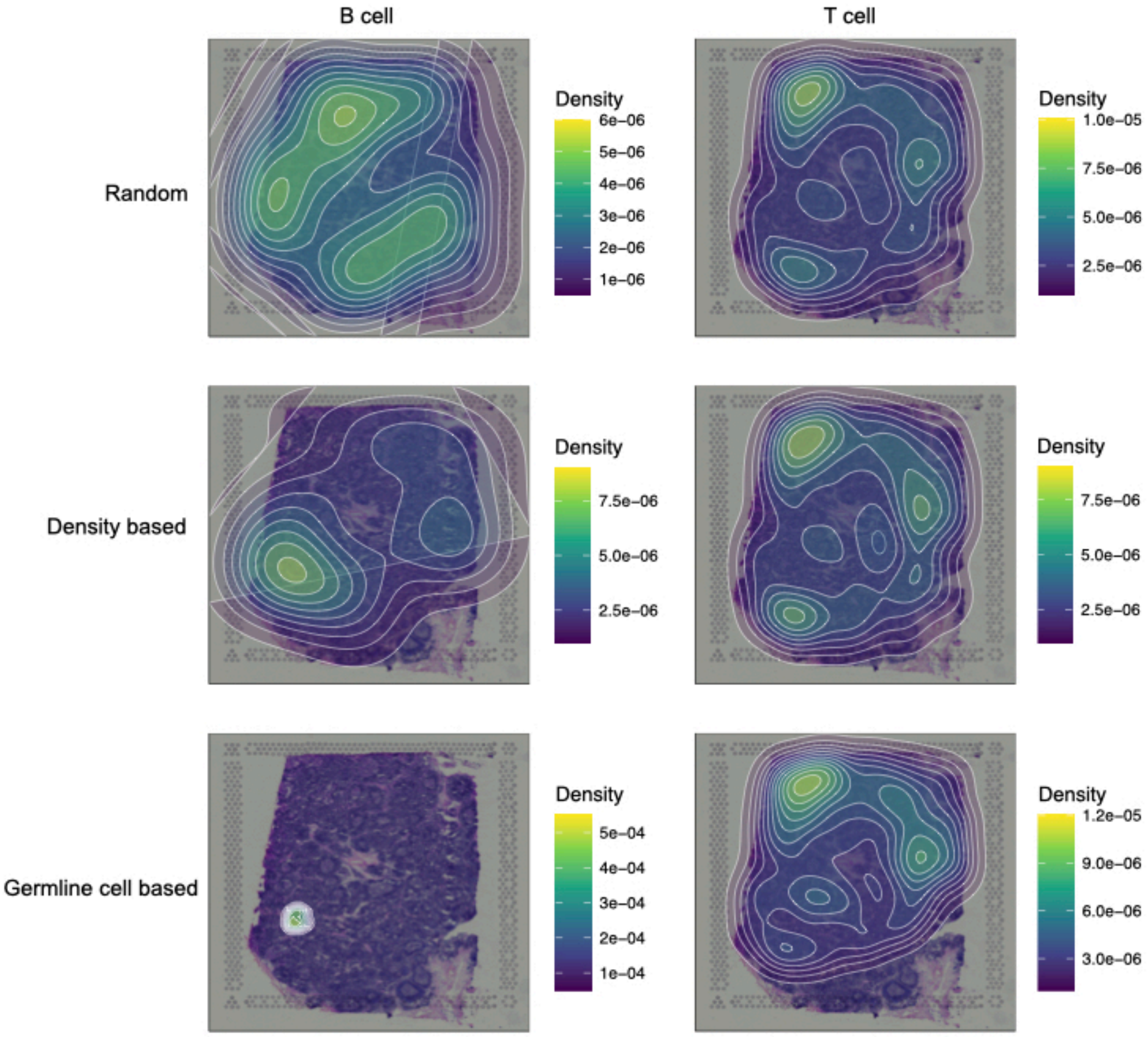

Figure S6. B and T cell density contour map of the most expanded clone when immune receptor sequences were assigned to cells in three different methods: random assignment, density-based assignment, and germline-cell-based assignment. Colors correspond to the calculated density values.

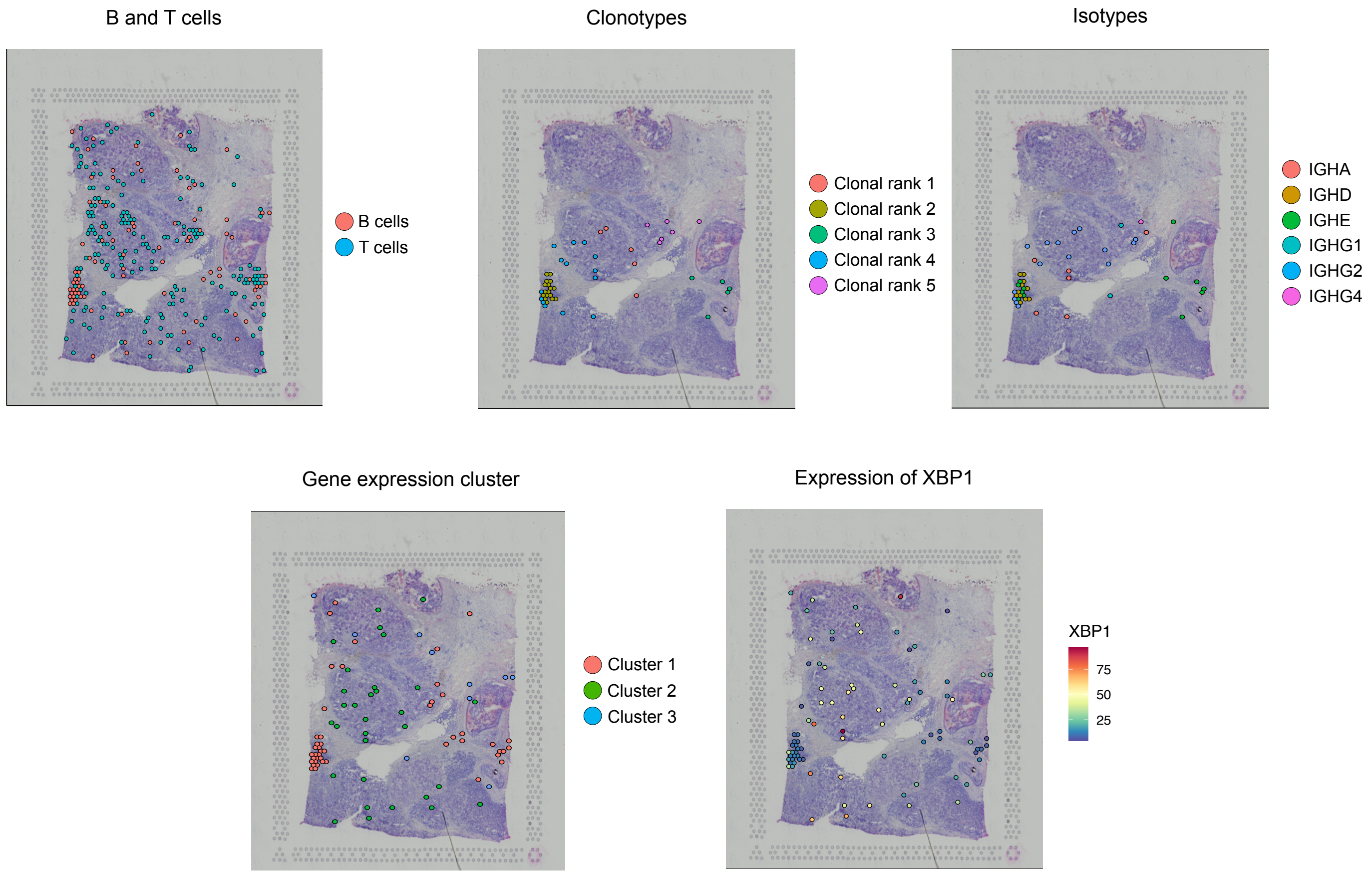

Figure S7. Spatial analysis on human breast cancer tissue by simulation. Cells were assigned with simulated VDJ sequences and clonal identity. 1) T cells and B cells identified based on marker gene expression. 2) B cell clonotypes ranked by number of cells in the clone. 3) B cells colored in isotypes. 4) B cells colored by gene expression clusters determined by UMAP. 5) B cells colored by gene XBP1 expression level.

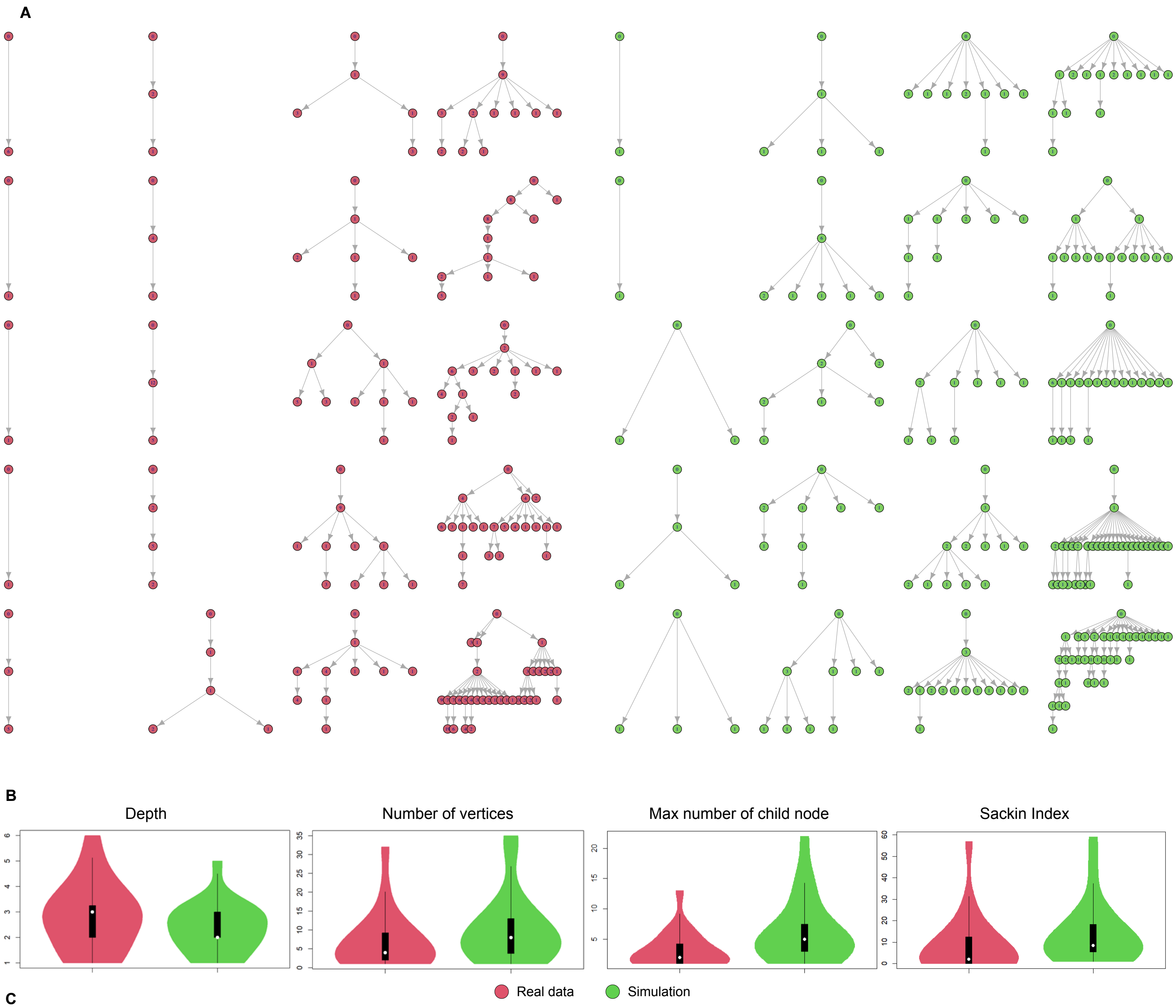

C

Metric	Real (Range)	Simulation (Range)
Network Depth	1~6	1~5
Number of vertices	2~33	2~36
Max number of child node	1~13	0~22
Sackin Index	0~57	1~59

Figure S8. Network metric analysis on real data and simulated data. A. 20 mutational networks built from real data and simulation ranked by size. Real networks were inferred based on sequence edit distance from germline sequence, taken from protein-immunized mouse bone marrow plasma cell repertoire. B. The Violin plots show the distribution of depth, number of vertices and maximum number of child node among 20 real networks and 20 simulated networks. The child node is defined as nodes that are derived from the same mother node, to represent the level of SHM within a network. Sackin index represents the level of imbalance of the tree, defined as the sum of the depth of all leaves. C. The table shows the range of these three metrics.

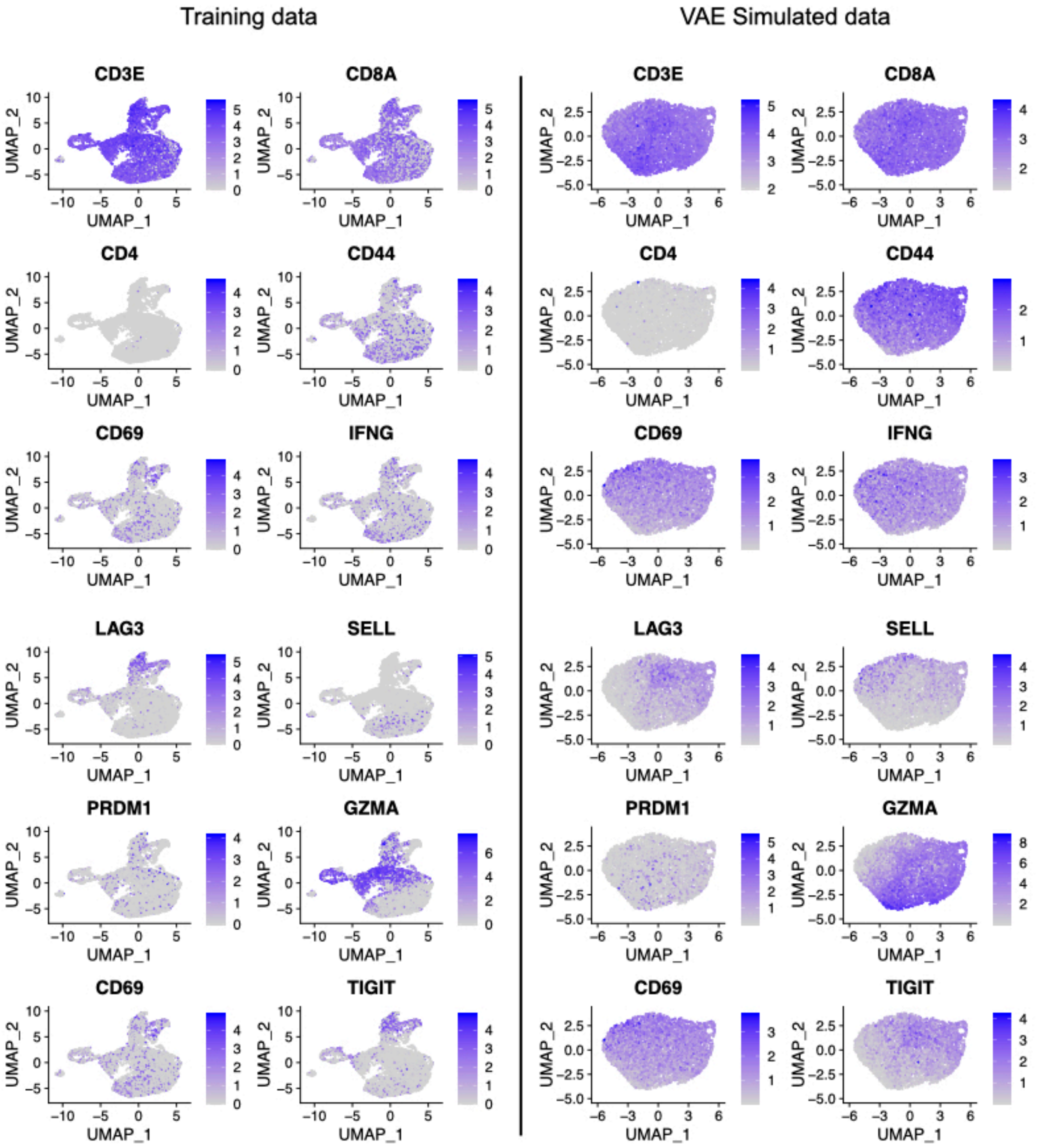

Figure S9. UMAP presentation of simulated gene expression data (right lane) comparing with training data (left lane). Cell populations with similar marker gene expression levels to real data are present in simulated data.
