# Supplementary figures and images for: Echidna: integrated simulations of single-cell immune receptor repertoires and transcriptomes
Source: Bioinform Adv. 2022 Sep 2;2(1):vbac062. doi: 10.1093/bioadv/vbac062 (PMC9710610; doi:10.1093/bioadv/vbac062)

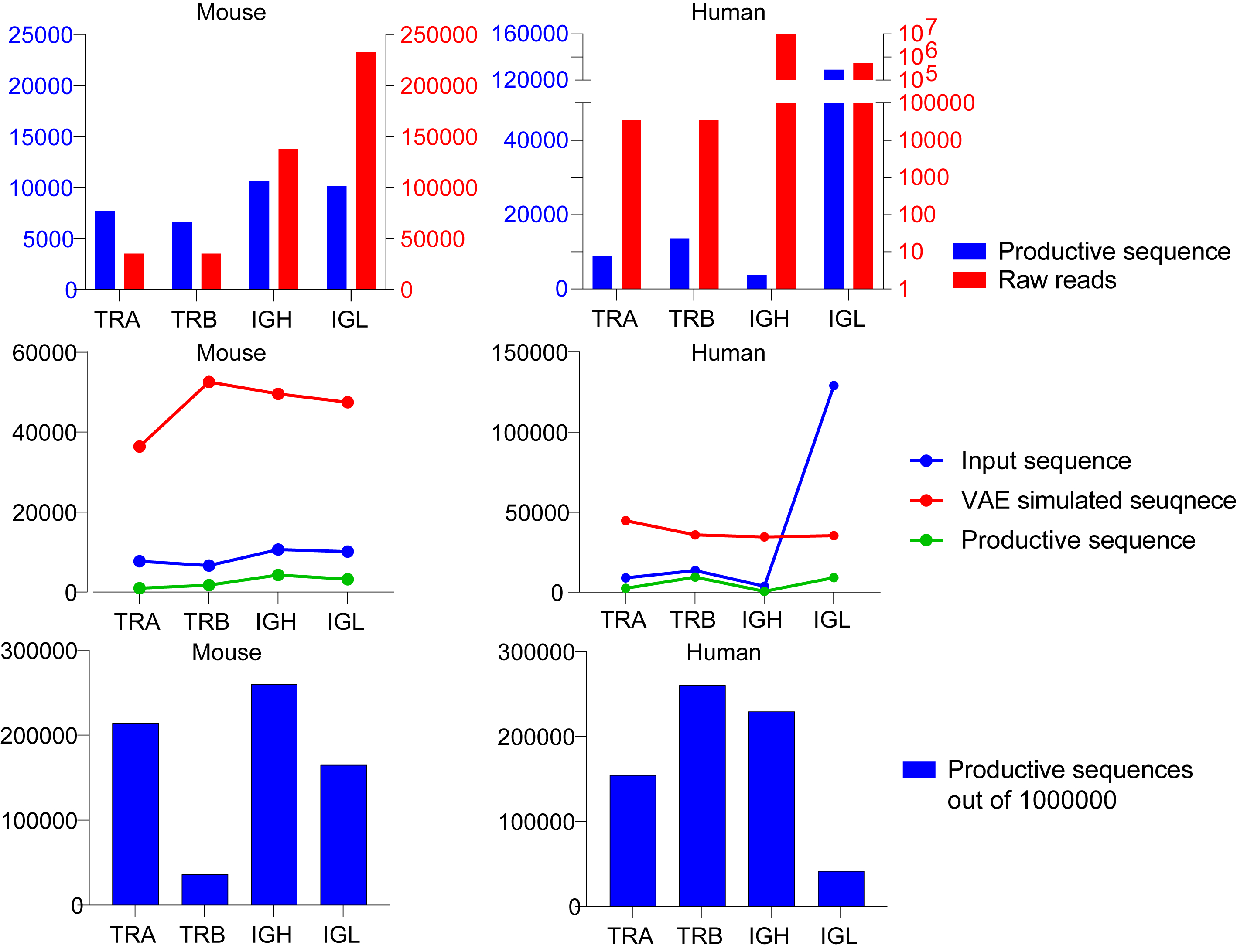

Supplement: vbac062_Supplementary_Data [file vbac062_supplementary_data.zip › S2.tiff]

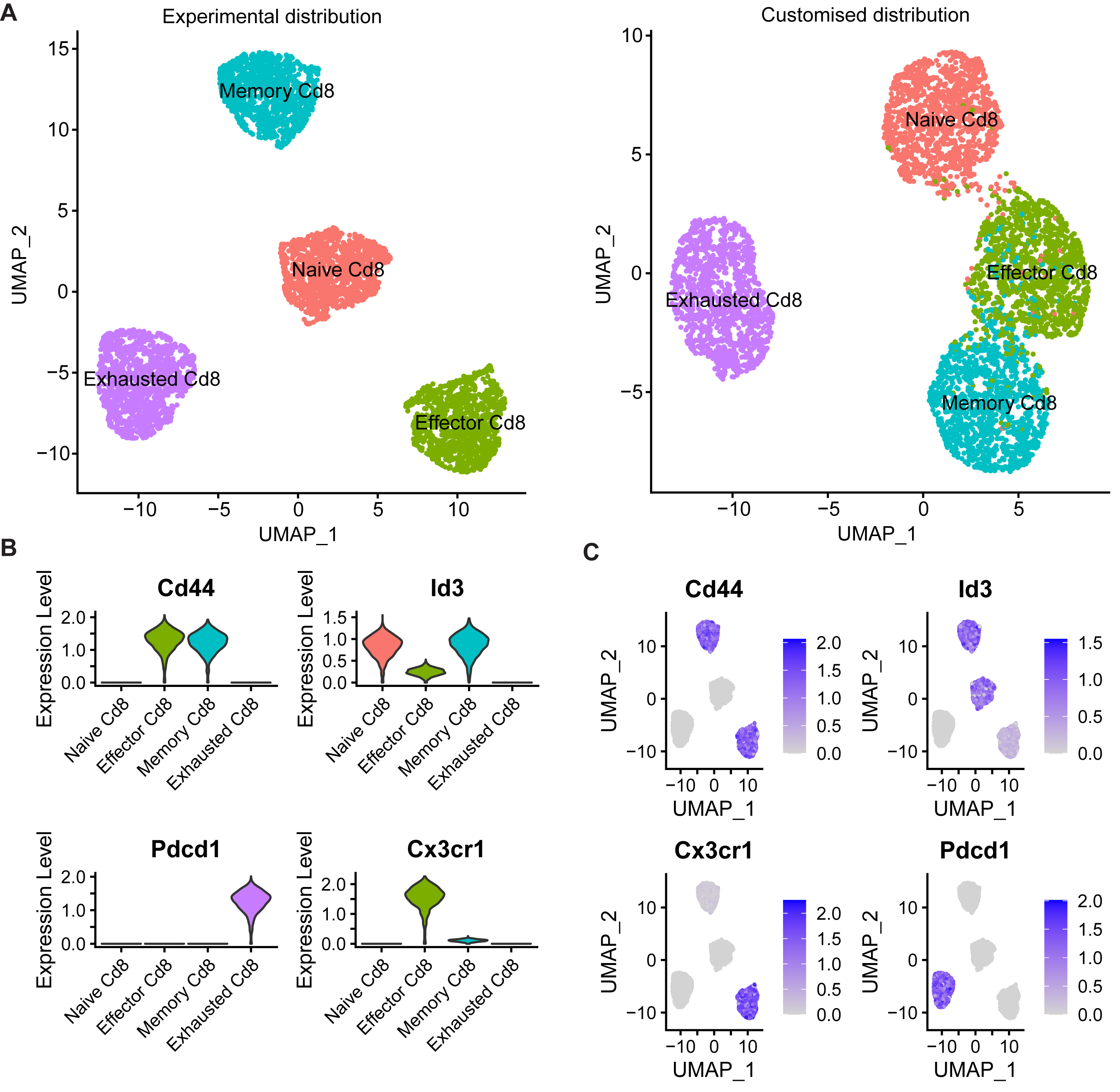

Supplement: vbac062_Supplementary_Data [file vbac062_supplementary_data.zip › S3.tiff]

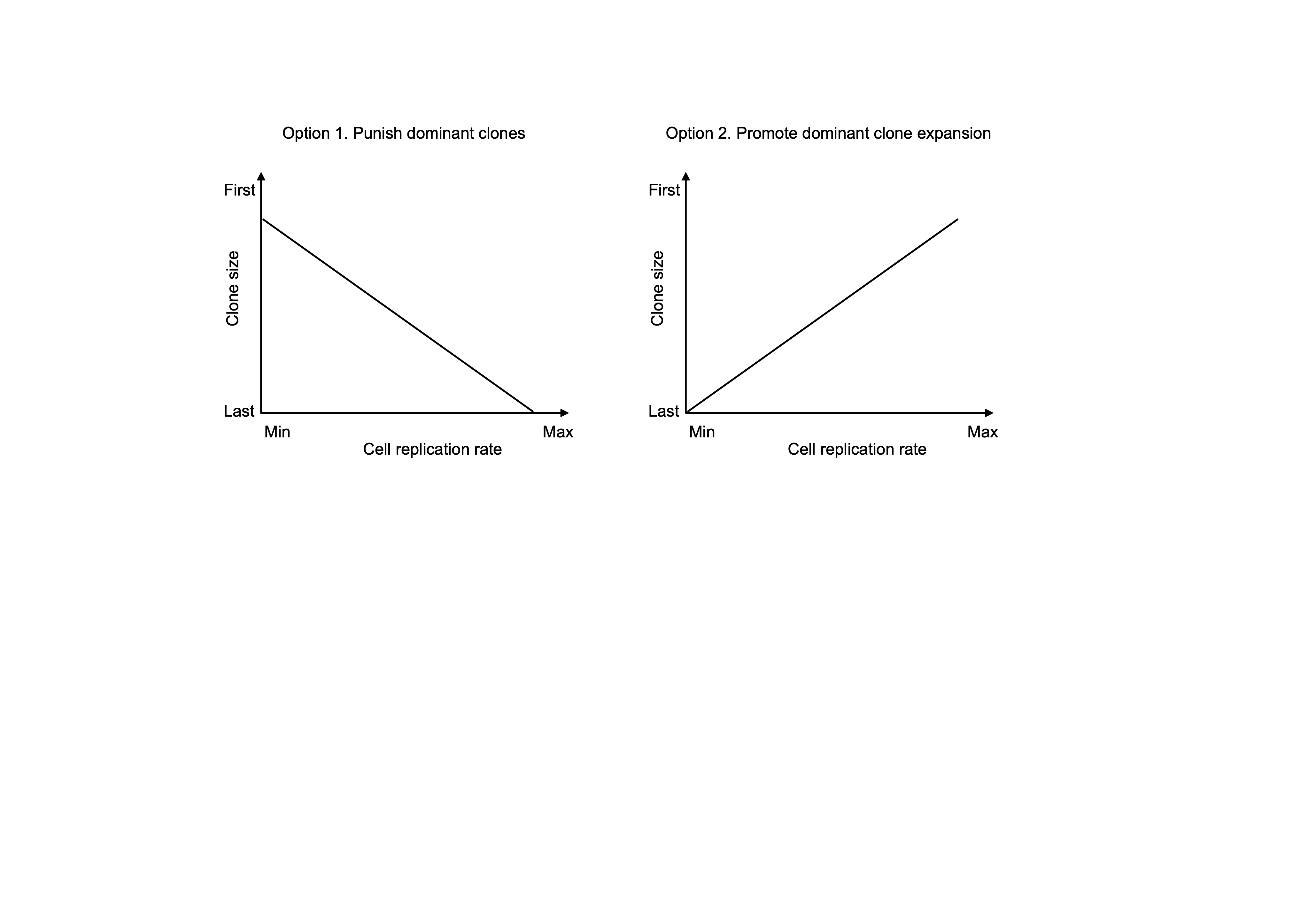

Supplement: vbac062_Supplementary_Data [file vbac062_supplementary_data.zip › S4.tiff]

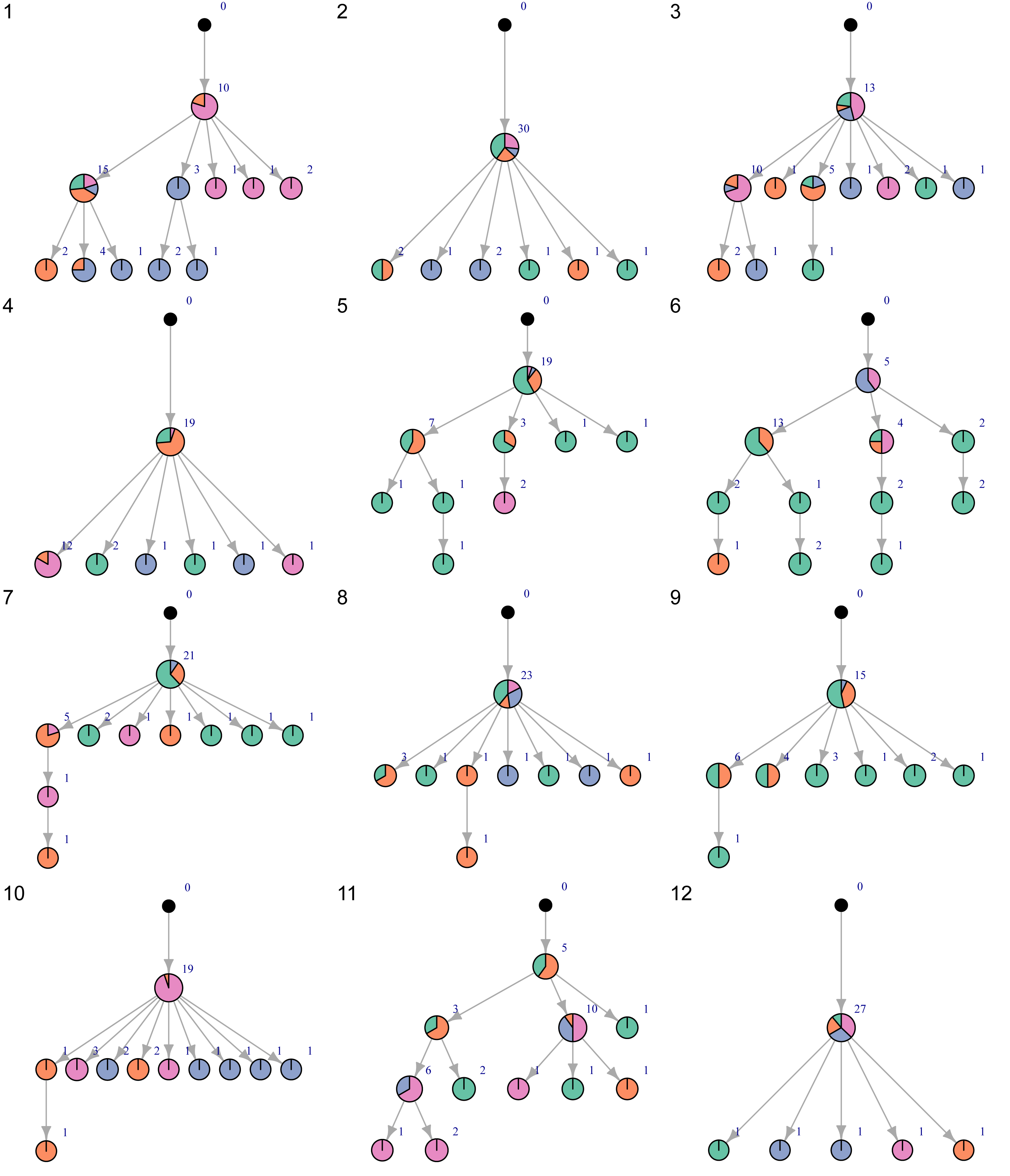

Supplement: vbac062_Supplementary_Data [file vbac062_supplementary_data.zip › S5.tiff]

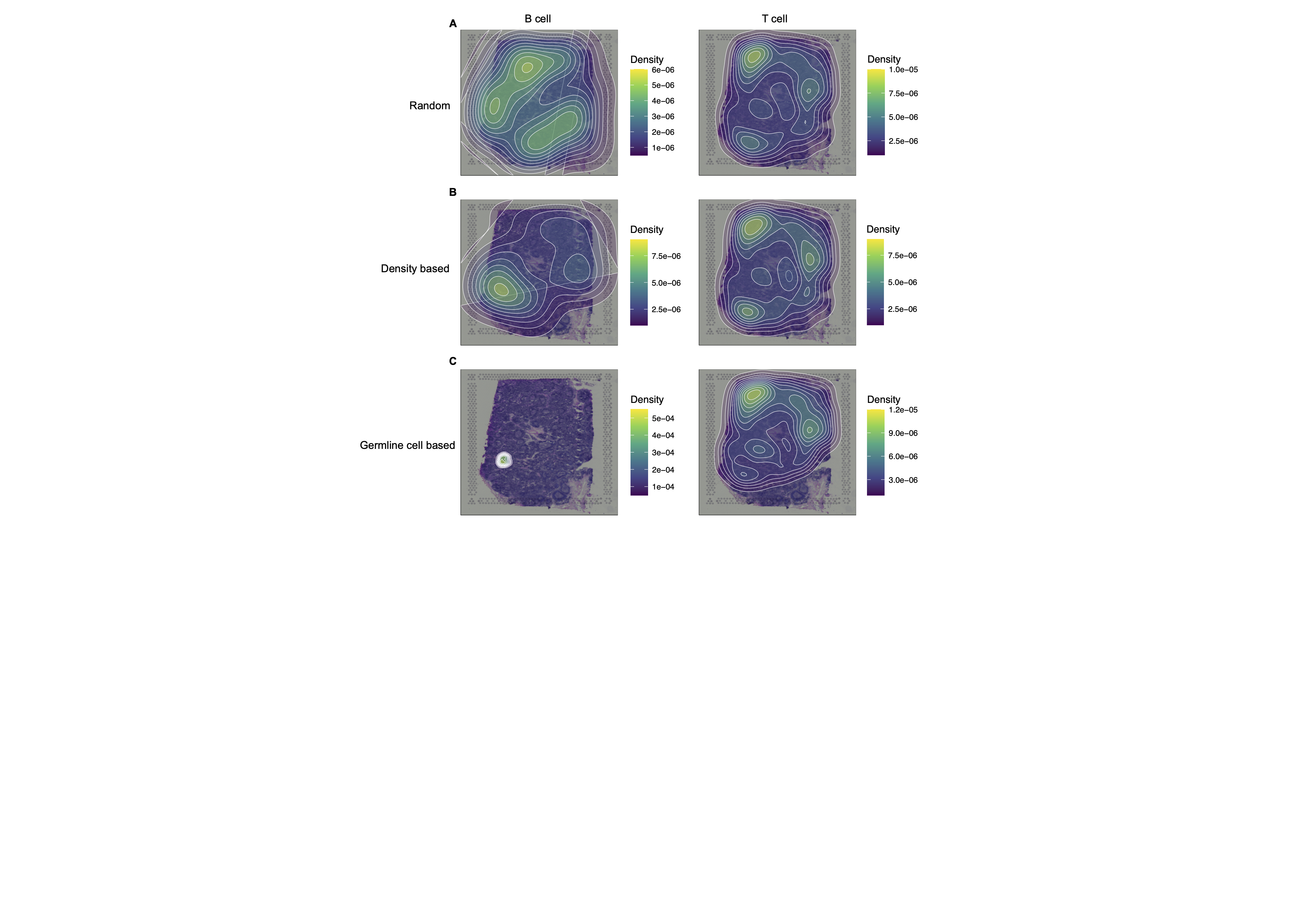

Supplement: vbac062_Supplementary_Data [file vbac062_supplementary_data.zip › S6.tiff]

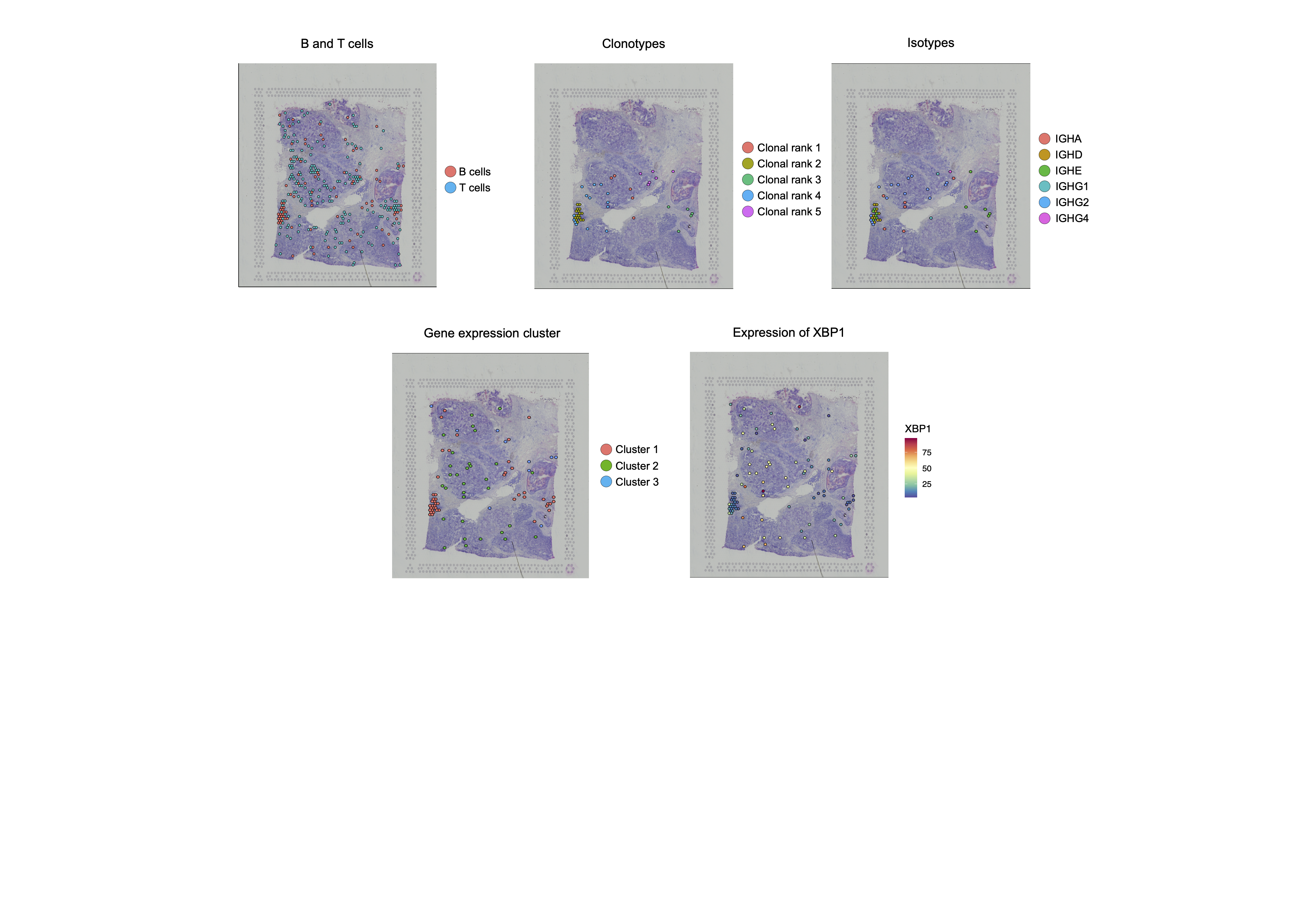

Supplement: vbac062_Supplementary_Data [file vbac062_supplementary_data.zip › S7.tiff]

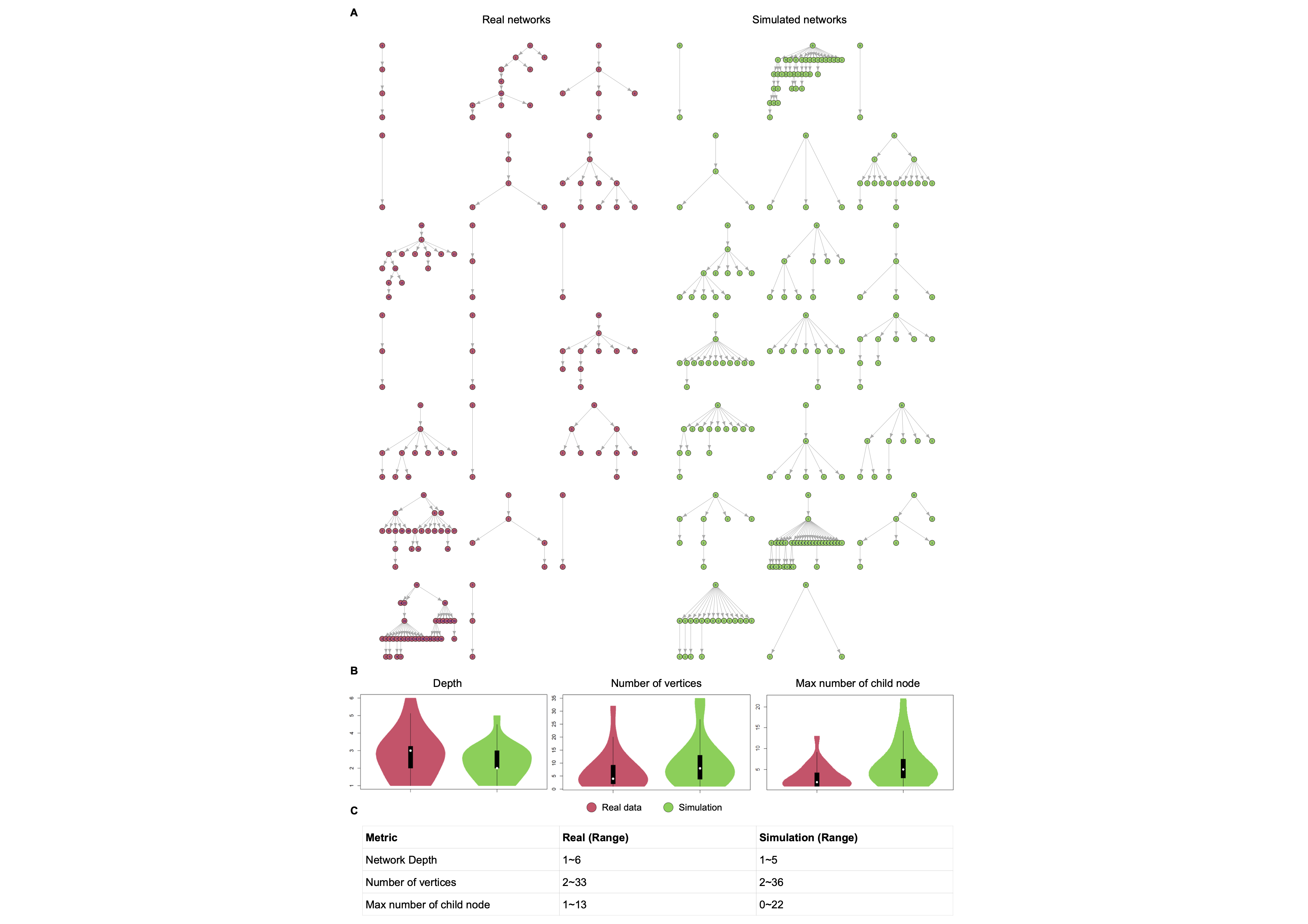

Supplement: vbac062_Supplementary_Data [file vbac062_supplementary_data.zip › S8.tiff]

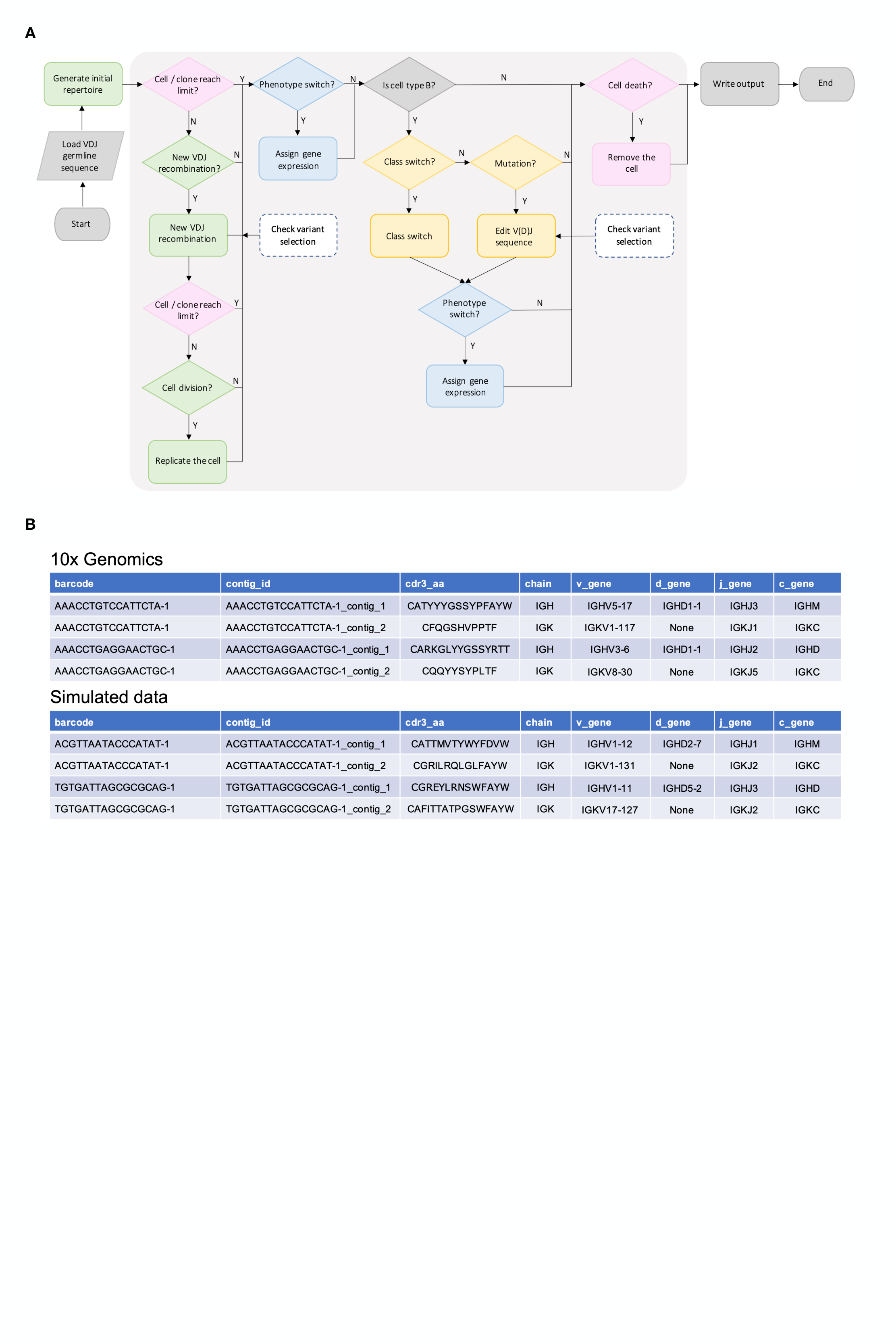

Supplement: vbac062_Supplementary_Data [file vbac062_supplementary_data.zip › S1.tiff]
